# Genome-Wide RNA Polymerase II Profiles and RNA Accumulation Reveal Kinetics of Transcription and Associated Epigenetic Changes During Diurnal Cycles

**DOI:** 10.1371/journal.pbio.1001442

**Published:** 2012-11-27

**Authors:** Gwendal Le Martelot, Donatella Canella, Laura Symul, Eugenia Migliavacca, Federica Gilardi, Robin Liechti, Olivier Martin, Keith Harshman, Mauro Delorenzi, Béatrice Desvergne, Winship Herr, Bart Deplancke, Ueli Schibler, Jacques Rougemont, Nicolas Guex, Nouria Hernandez, Felix Naef, the CycliX consortium

**Affiliations:** 1 Department of Molecular Biology, University of Geneva, Geneva, Switzerland; 2 Center for Integrative Genomics, Faculty of Biology and Medicine, University of Lausanne, Lausanne, Switzerland; 3 The Institute of Bioengineering, School of Life Sciences, Ecole Polytechnique Fédérale de Lausanne, Lausanne, Switzerland; 4 Vital IT, Swiss Institute of Bioinformatics, Lausanne, Switzerland; 5 Département de Formation et de Recherche, Centre Hospitalier Universitaire Vaudois and University of Lausanne, Lausanne, Switzerland; 6 Bioinformatics and Biostatistics Core Facility, School of Life Sciences, Ecole Polytechnique Fédérale de Lausanne, Lausanne, Switzerland; Charité - Universitätsmedizin Berlin, Germany

## Abstract

Interactions of cell-autonomous circadian oscillators with diurnal cycles govern the temporal compartmentalization of cell physiology in mammals. To understand the transcriptional and epigenetic basis of diurnal rhythms in mouse liver genome-wide, we generated temporal DNA occupancy profiles by RNA polymerase II (Pol II) as well as profiles of the histone modifications H3K4me3 and H3K36me3. We used these data to quantify the relationships of phases and amplitudes between different marks. We found that rhythmic Pol II recruitment at promoters rather than rhythmic transition from paused to productive elongation underlies diurnal gene transcription, a conclusion further supported by modeling. Moreover, Pol II occupancy preceded mRNA accumulation by 3 hours, consistent with mRNA half-lives. Both methylation marks showed that the epigenetic landscape is highly dynamic and globally remodeled during the 24-hour cycle. While promoters of transcribed genes had tri-methylated H3K4 even at their trough activity times, tri-methylation levels reached their peak, on average, 1 hour after Pol II. Meanwhile, rhythms in tri-methylation of H3K36 lagged transcription by 3 hours. Finally, modeling profiles of Pol II occupancy and mRNA accumulation identified three classes of genes: one showing rhythmicity both in transcriptional and mRNA accumulation, a second class with rhythmic transcription but flat mRNA levels, and a third with constant transcription but rhythmic mRNAs. The latter class emphasizes widespread temporally gated posttranscriptional regulation in the mouse liver.

## Abstract 2

Genome-wide rhythms in RNA polymerase II loading and dynamic chromatin remodeling underlie periodic gene expression during diurnal cycles in the mouse liver.

## Author Summary

In mammalian organs such as the liver, many metabolic and physiological processes occur preferentially at specific times during the 24-hour daily cycle. The timing of these rhythmic functions depends on a complex interplay between the endogenous circadian clock and environmental timing cues relayed through the master circadian clock in the suprachiasmatic nucleus, or via feeding rhythms. These rhythms can be implemented on several regulatory levels, and here we aimed at a better understanding of the transcriptional and epigenetic changes that regulate diurnal rhythms. We performed genome-wide analysis of the locations of RNA polymerase II (Pol II) and the epigenetic histone modifications H3K4me3 and H3K36me3 at specific times of day, relating these data to mRNA expression levels. Our analyses show that Pol II transcriptional rhythms are biphasic in mouse liver, having predominant peak activities in the morning and evening. Moreover, dynamic changes in histone marks lag transcription rhythms genome-wide, indicating that the epigenetic landscape can be remodeled during the 24-hour cycle. Finally, a quantitative analysis of temporal Pol II and mRNA accumulation profiles indicates that posttranscriptional regulation significantly contributes to the amplitude and phase of mRNA accumulation profiles. While many studies have analyzed how transcription and chromatin states are modified during irreversible cell differentiation processes, our work highlights how these states can evolve reversibly in a system exhibiting periodicity in time.

## Introduction

In most mammalian organs, widespread diurnal rhythms in mRNA expression underlie the temporal compartmentalization of cellular and physiological processes [1]. In liver, these rhythms reflect the combined effects of regulation by time-varying systemic signals and by the autonomous circadian oscillator [2],[3]. Pol II–dependent gene expression in the liver exhibits widespread and large-amplitude daily rhythms in transcript accumulation that temporally compartmentalize key hepatic functions [4] such as lipid and carbohydrate metabolism [5],[6], sterol biosynthesis [7], or detoxification [8]. In liver, chromatin immuno-precipitation (ChIP) studies on key circadian transcription factors are shedding light on how the clock achieves phase-specific transcription rhythms [9]–[14]. Studies on many individual genes have shown that circadian transcription is accompanied by temporally varying epigenetic modifications [9],[15]–[19], but it is only recently that this question is being addressed genome-wide [20]. Such studies are important as they can reveal the relationships between the kinetics of transcription and related histone modifications in a system that returns to the same state every 24 h.

Here we quantified in time the recruitment of Pol II to gene promoters, the loading of Pol II in gene bodies, the levels of H3K4 and H3K36 trimethylation, as well as the accumulation of mRNAs at six time points around a 24-h cycle. We mapped H3K4 and H3K36 trimethylation because these marks are known to accumulate to high levels just downstream of active TSSs in the first case [21]–[23], and within the body of transcribed genes, with higher accumulation toward the end of the transcription unit, in the second case [21],[24],[25]. Comparison of these marks with Pol II accumulation provides us with two independent ways to assess location of active TSSs and transcription units. We used these data to provide a comprehensive view on how these quantities are dynamically related in terms of phases and amplitudes. Moreover, a mathematical model relating transcription and mRNA accumulation profiles, which enforces the associated quantitative constraints on phases and amplitudes, reveals a role for posttranscriptional processes in cyclic mRNA accumulation [26].

## Results

### Temporal ChIP-Seq Profiles for Pol II, H3K4me3, and H3K36me3

**Figure 1:**
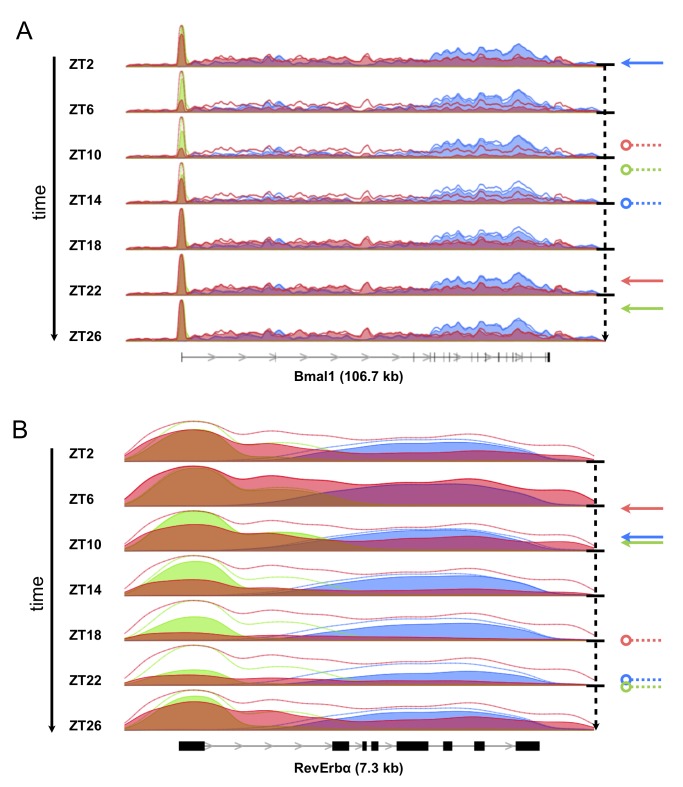
Pol II, H3K4me3, and H3K36me3 genomic profiles of core circadian clock genes measured around the clock. (A) The density profiles of Pol II (red), H3K4me3 (green), and H3K36me3 (blue) are indicated for the Bmal1 gene, which spans 107 kb on chromosome 7, with the thin lines above the profiles indicating the position-specific temporal maxima. The gene structure (RefSeq transcripts) is shown below the panel. The dashed lines starting with a circle and the arrows represent minima and maxima, respectively, of gene body Pol II occupancy (red), promoter H3K4me3 occupancy (green), and gene body H3K36me3 occupancy (blue), as estimated by cosine fits (Materials and Methods). Maximal Pol II, H3K4me3, and H3K36me3 densities are reached at ZT21, ZT23, and ZT2. (B) As in (A), but for the *RevErbα* (*Nr1d1*) gene, which spans 7.3 kb on chromosome 11. Maximal Pol II, H3K4me3, and H3K36me3 densities are reached at ZT6, ZT9, and ZT9. Temporal animations of these profiles are provided as supplemental movies. Similar profiles for the circadian *Per1* gene and constitutive *Tbp* gene are shown in Figure S1.

Analysis of ChIP-seq profiles for Pol II (RPB2 subunit), H3K4me3, and H3K36me3 in mouse liver revealed that Pol II occupancy correlated well with known transcription phases of core clock and clock-control genes (Figures 1 and S1). For example, the master clock regulator *Bmal1* was maximally occupied by Pol II, both at the promoter and within the gene body, at Zeitgeber Time (ZT) 22 (ZT0 corresponds to light on and ZT12 to light off), whereas its direct target and repressor, the nuclear receptor *RevErbα*, reached peak occupancy levels at ZT6 (Figure 1A,B), consistent with the known temporal transcription pattern of these genes [27]. The temporal variation of these profiles indicates that Pol II is loaded dynamically to the gene promoters and that productive elongation as reflected by Pol II occupancy in the gene bodies varies in phase with the promoter signal (Figure 1 and Movies S1, S2). In addition, these promoters showed localized signals of H3K4me3, whose levels were above background at all times and cycled with a slight delay compared to Pol II occupancy (Figure 1). As expected, the H3K36me3 mark, reflecting active transcription and splicing [28],[29], was found within gene bodies, with typically higher levels towards the 3′-proximal end of genes [24],[25]. H3K36me3 levels cycled with a delayed phase and with damped amplitude compared to H3K4me3 and Pol II occupancy.

### The Spatial Organization of Pol II and Associated Chromatin Modifications Along the Genome

**Figure 2:**
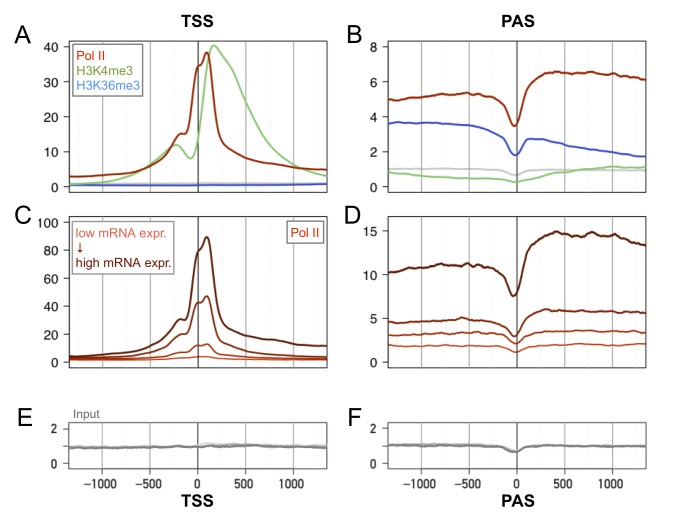
Genomic profiles of Pol II, H3K4me3, and H3K36me3 densities around transcription start sites (TSSs) and polyadenylation sites (PASs) at ZT2. (A) Average signals over 11,217 genes with nonoverlapping TSSs (see Materials and Methods) for each mark around the TSSs: Pol II (red), H3K4me3 (green), H3K36me3 (blue), and input chromatin (gray). (B) As in (A) but around the PAS. (C) Average Pol II signals over transcripts split by quartile, based on the level of expression as measured by microarrays. Each quartile is represented by a distinct color shading from light (lowest quartile in mRNA expression) to dark (upper quartile in mRNA expression). (D) As in (C) but for the PAS. (E–F) Profiles of input chromatin. Note that the depletion at the PAS only partially explains the dips in panels (B) and (D). Vertical axes have arbitrary units, but the scales on the left and right panels can be compared for the same marks.

Pol II transcription starts with recruitment of the polymerase complex to gene promoters to form closed complexes. This initial step is followed by the isomerization to the open complex, abortive transcription, promoter escape, release from possible proximal pausing, elongation, and transcript termination (reviewed in [30],[31]). The transition kinetics from one phase to the next can lead to distinct genomic Pol II occupation profiles [32]. We analyzed the Pol II spatial profiles around transcript start sites (TSSs) and polyadenylation sites (PASs) (Figure 2A,B). Near TSSs, we obtained profiles consistent with previous studies [21],[33], although high read coverage enabled us to resolve three bumps located approximately at positions −200, +1, and +80 bp. The Pol II signal trailed downstream of the TSS, reflecting productive elongation [32],[34]. At PASs, we observed a dip in the Pol II signal followed by an increase above the level observed upstream of the PASs, suggesting a slowing down of the polymerase after transcript cleavage and before transcription termination [31],[32],[34]. The H3K4me3 mark peaked about 200 nucleotides downstream of the TSS [21],[35] and was absent around the PAS. In contrast, the H3K36me3 mark was absent near the TSS but accumulated increasingly toward the 3′-proximal end of genes [24] to dip just before the PAS, rebound, and then slowly decrease (Figure 2A,B). We then stratified our data into quartiles according to mRNA accumulation levels obtained from the same samples. Pol II occupancy as well as the two histone marks clearly scaled with mRNA accumulation (Figures 2C,D and S2A–D), and Pol II occupancy scaled with increased CpG content in promoters (Figure S2E,F). This suggests that in mouse liver, the genes most highly occupied by Pol II are in general genes with promoter-associated CpG islands.

### Promoter Proximal Pol II Occupancy Is Rhythmic in Liver and Predicts Transcription

**Figure 3:**
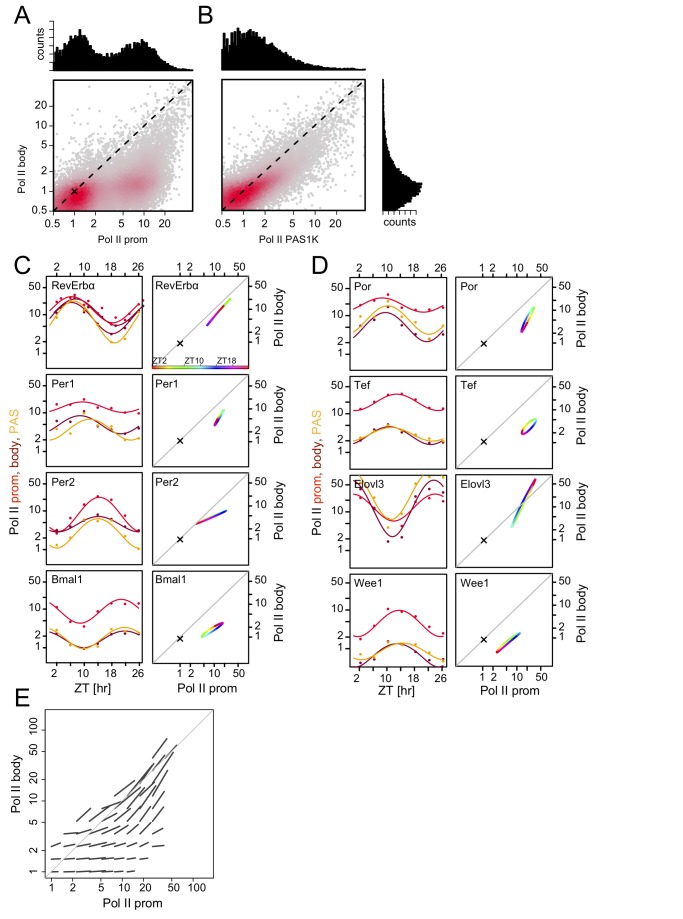
Temporal Pol II occupancies at promoters and in gene bodies oscillate with similar phases. (A) Pol II occupancy at ZT2 in promoters (mean in ±1 kb regions around TSSs) versus gene bodies (mean over the regions from +1 kb to the PAS) for all genes in logarithmic scale. Color intensity indicates population density. One transcription unit per gene is shown (the selection is based on H3K4me3 and Pol II signals at promoters and PAS, as described in Materials and Methods). Two main populations can be distinguished: one with low Pol II occupancy in both promoter and gene body regions (lower left cloud), corresponding to silent or weakly transcribed genes, and one with higher Pol II occupancy within promoter regions and, to a lower extent, gene body regions (fainter cloud shifted slightly up and to the right). The bimodality of the promoter signal is clearly seen in the projection (histogram above the horizontal axis), whereas the signal in the gene body (elongating polymerases) has a lower dynamic range (histogram on the vertical axis shown in panel B). The cross sign, also shown in panels C and D, indicates background levels estimated from the lower maxima of the histograms. (B) Pol II occupancy at ZT2 in the first 1 kb window after the PAS (PAS1K) versus gene bodies (as in A). The two measures show high correlation, but PAS1K has a larger dynamic range (see Figure 2B). In (A) and (B), data are shown for ZT2, but all time points looked virtually identical. (C and D) Temporal profiles of Pol II promoter/gene body occupancy ratios for core clock genes (C) and selected output genes (D). Left, temporal profiles for promoters (red), gene bodies (brown), and PAS (orange) together with cosine fits. Right, the data from the left panels for the promoter and gene body are plotted against each other in the coordinate system of panel A. ZT times are color-coded (see color bar). Cross signs indicate background levels. Note logarithmic scale on axes. (E) Genome-wide analysis showing that Pol II occupancies at promoters and in gene bodies co-vary in time. Each line shows the average orientation and amplitude of changes during a diurnal cycle for genes in regions on a grid. The nonbinned plot is shown in Figure S3C.

The results above reveal the spatial arrangement of Pol II, H3K4me3, and H3K36me3 along genes as well as the dynamic changes of these marks during a diurnal cycle. We further analyzed the genome-wide relationship between Pol II density at promoters, probably reflecting polymerases with the C-terminal domain (CTD) either hypophosphorylated or phosphorylated on Serine (Ser) 5 in the closed complex, open complex, and paused states, and that of Pol II density within gene bodies, likely to represent elongating enzymes with CTDs increasingly phosphorylated on Ser 2 in addition to Ser 5 (see [36] and [34] and references therein). We found two main populations of genes: those with low Pol II in both promoters and gene bodies and those with higher Pol II in both regions (Figure 3A). Our mRNA expression data indicated that the first group corresponds to genes transcribed at background levels and the second to actively transcribed genes (Figure S3B). The lower Pol II density observed in gene bodies relative to promoter regions (by a factor of 3 to 10; Figure 3A) likely reflects polymerases elongating at a typical speed of ∼60 nt/s [37], thus diluting the read signals as compared to the nearly static polymerases proximal to promoters. The Pol II densities in the gene bodies and downstream of the PAS correlated well; nevertheless, the latter showed an increased signal compared to the former, perhaps due to Pol II pausing downstream of the PAS (Figures 2B and 3B) [32],[38].

The net transcription rate can be controlled on several levels: at the level of Pol II recruitment to promoters by transcription factors as studied in vitro [38] as well as at a postrecruitment stage through the release of Pol II from promoter proximal pausing [24]. The relative importance of these two regulation steps seems to vary in different biological settings [34]. Our time series allowed us to examine the temporal relationships between Pol II occupancy in promoters, in gene bodies, and downstream of the PASs. For both core clock (Figure 3C) and circadian output genes (Figure 3D), we found that, at the measured time resolution, the three quantities varied in phase. This is seen both in the overlaid temporal profiles (left rows in Figure 3C,D) and in the temporally correlated promoter and gene body signals (right rows in Figure 3C,D). The tendency of Pol II occupancy in promoters and gene bodies to vary in phase was confirmed genome-wide as indicated by the averaged orientation of temporal promoter and gene body signals (Figures 3E and S3D–G) and similarly for the relationship between promoter and PAS signals (Figure S3H). As supported by a simulation of a mathematical model of transcription describing the Pol II recruitment step, isomerization to the open complex, promoter escape, release from promoter proximal pausing, and elongation (Figure S4), this argues that rhythmic loading of Pol II to gene promoters, rather than a rhythmic transition from a paused state to productive elongation, primarily underlies diurnal transcription rhythms for the vast majority of transcripts expressed above background levels. Indeed, the scenario of a temporally regulated pausing release would lead to anticorrelated promoter and gene body signals traces (Figure S4), which is not observed.

### The Chromatin Landscape Is Dynamically Remodeled During Diurnal Cycles

**Figure 4:**
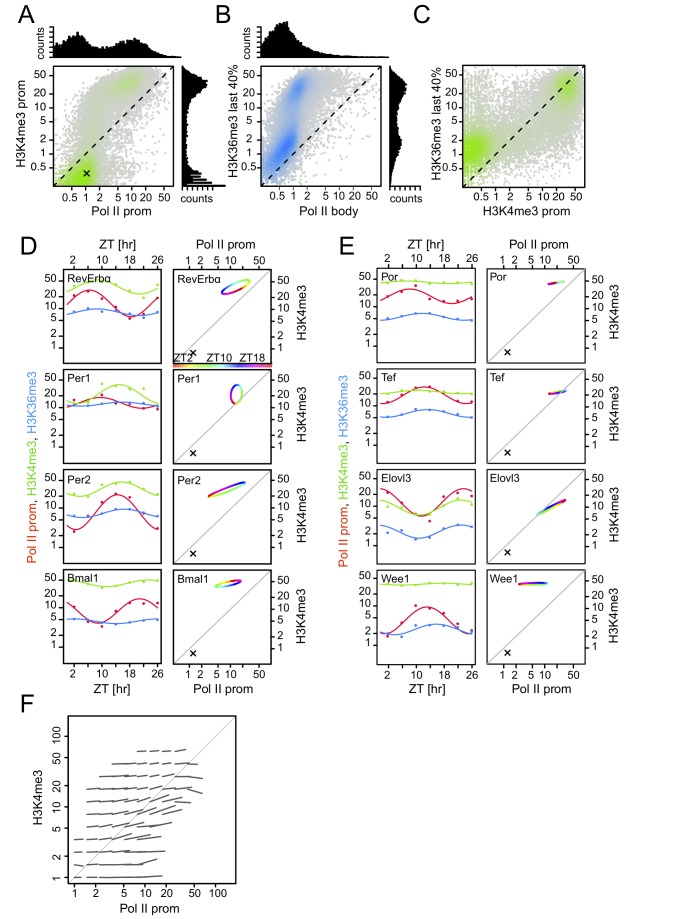
H3K4me3 and H3K36me3 marks vary during the diurnal cycle with reduced amplitude as compared to Pol II occupancy. (A) H3K4me3 promoter levels versus Pol II promoter occupancy at ZT2. Two populations can be identified from the densities: silent (or weakly active) promoters (lower left cloud) and active promoters (fainter cloud shifted above the diagonal and to the right). Bimodality in both signals is clearly seen in the projections (histograms). The cross sign, also shown in panels D and E, indicates background levels estimated from the lower maxima of the histograms. (B) H3K36me3 levels (quantified over the most 3′-proximal 40% of gene bodies) versus Pol II body occupancy at ZT2. Two populations can be identified from the densities: silent (or weakly transcribed) genes (lower left cloud) and transcribed genes. (C) H3K36me3 levels as in (B) versus H3K4me3 promoter levels at ZT2. This comparison shows the two classes most clearly, indicating that the large majority of genes harboring H3K4me3 marks are transcribed. In (A–C), data are shown for ZT2, but all time points looked identical. (D–E) Temporal profiles of H3K4me3 and H3K36me3 marks, and promoter Pol II occupancy for some core clock genes (D) and selected output genes (E). Left, temporal profile for promoter Pol II occupancy (red), H3K4me3 marks (green), and H3K36me3 marks (blue) together with cosine fits. Right, the cosine fits for Pol II promoter occupancy and H3K4me3 plotted against each other in the coordinates of panel A. ZT times are color-coded (see color bar). Crosses indicate background levels. Note that levels of H3K4me3 remain relatively high at the troughs of transcription (as measured by Pol II density). (F) Genome-wide temporal analysis showing that H3K4me3 modifications at promoters show compressed amplitudes compared to Pol II promoter occupancy (compare with Figure 3E). Each line shows the average orientation and amplitude of changes during a diurnal cycle for genes in regions of a grid. The nonbinned plot is shown in Figure S5.

A similar analysis of the levels of Pol II and H3K4me3 at gene promoters identified two well-separated populations exhibiting either both low H3K4me3 and Pol II levels or high H3K4me3 and Pol II levels (Figure 4A). Most likely, H3K4me3 separates nonactive from active genes in the liver, consistent with previous data [21]–[23],[39],[40]. Similarly, the comparison of H3K36me3 with Pol II in gene bodies, both indicative of active transcription, revealed that the H3K36me3 signals split into populations with low and high gene body Pol II occupancy, probably reflecting non- or lowly transcribed and actively transcribed genes (Figure 4B). This was even more obvious when H3K36me3 was compared with Pol II density at promoters (Figure S3A), again reflecting the lower Pol II sampling depth in gene bodies. Comparing H3K4me3 at promoters and H3K36me3 in gene bodies (Figure 4C) showed that the populations identified by either mark were highly correlated, indicating that, in the liver, H3K4me3-marked promoters are often actively transcribed. While this holds strictly for constitutively transcribed genes, rhythmically transcribed genes are among the exceptions as the promoters of both core clock (Figure 4D) and clock output genes (Figure 4E) harbored relatively high levels of H3K4me3 even at times of lowest Pol II occupancy. Thus the amplitude of the H3K4me3 rhythm was damped compared to that of Pol II. For example, Pol II occupancy for the *Per2* gene dropped close to background levels at nadir time ZT2, whereas H3K4me3 levels remained relatively high (Figure 4D–F). H3K36me3 rhythms exhibited a similarly compressed cycle (Figures 4D,E and S5C,D). The compressed amplitudes of the methylation mark levels as compared to Pol II occupancy suggest firstly that histone modifications may saturate before maximal transcription is reached and secondly that in a system where transcription levels oscillate on the scale of 24 h, methylation marks may not be removed completely before transcription increases again. Thus, in a situation of fast changing transcription, methylation marks do not provide linear measures of transcription.

### Pol II Transcription Is Biphasic and Histone Modifications Are Delayed Compared to Transcription

**Figure 5:**
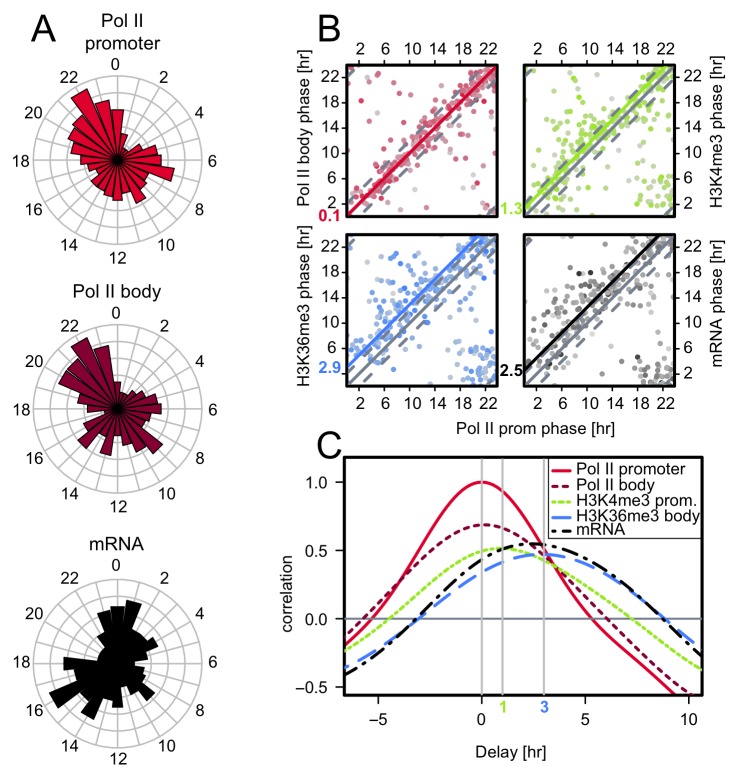
Temporal relationships of Pol II, H3K4me3, H3K36me3 profiles, and mRNA accumulation in mouse liver. (A) Phase histograms for cyclic genes. A selection of 284 genes (*p*<0.004, FDR = 0.3) showing cyclic patterns in all marks (see Materials and Methods) were fitted with a cosine function and the phase (peak time of the fit) was computed. These phases show a bimodal distribution for Pol II occupancy in promoters and gene bodies with maxima around ZT9 and ZT21, as well as in mRNA accumulation with a phase delay of approximately 3 h. (B) Phases for the same genes are represented in pairs, with color shade indicating *p* value (lower *p* values are darker) for the 24-h rhythm of the Pol II promoter signal. Relative to the phase of Pol II in promoters, we find high concordance for Pol II occupancy phases in gene bodies, an average delay of 1.3 h for H3K4me3 phases, and more spread H3K36me3 and mRNA phases with an average delay of about 3 h. Colored lines are mean-square regressions with intercepts corresponding to the average delays, as indicated in color. The thin dashed lines indicate ±2 h delays. (C) Temporal cross-correlation analysis. Using the same gene selection, we applied Fourier interpolation to obtain a continuous time trace (see Figure 3C,D and Figure 4D,E) and computed average cross-correlations between each mark and the corresponding Pol II promoter trace. Pol II occupancies in promoters and gene bodies are well-correlated and simultaneous, and H3K4me3 lags by about 1 h on average, whereas mRNA and H3K36me3 are phase-delayed by about 3 h. The same figure is shown for a more permissive selection (Figure S8, *n* = 752, *p*<0.018, FDR = 0.5).

We next aimed at studying the temporal relationships between Pol II transcription, histone marks, and mRNA accumulation. While the statistical power for rhythmicity analysis on individual datasets is limited with our low sampling [41],[42], we exploited the possibility to combine the rhythmicity scores of five different features (Pol II promoter, Pol II body, H3K4me3, H3K36me3, and mRNA) to define a set of transcripts that showed significant combined rhythms suitable for a global analysis of phase relationships (*n* = 284, *p*<0.004, Fisher's combined probability test, FDR = 0.3). Even at this permissive false discovery rate (FDR), this set shows excellent overlap with previously published transcriptome data (Figure S6A,B; Table S4) [41],[43], and an extended selection (*n* = 752) shows equally convincing overlap (Figure S6C–E). These genes clearly showed a bimodal distribution in peak Pol II phase with maxima at ZT9 and ZT21 both for the promoter and gene body signals (Figure 5A), consistent with models of phase-specific circadian transcription in which E-box elements drive the transcription of morning genes and RORE elements that of evening genes [44]. In fact, the transcription phases of promoter found to be bound by the E-box activator BMAL1 [11] and the repressor of RORE elements RevErbα [14] support this interpretation (Figure S7A). Since we found a strong correlation between Pol II loadings at promoters and CpG content (Figure S2E), we stratified our analysis accordingly. While the phase distributions did not show a significant difference in promoters with low versus high CpG content (Figure S7B), the rhythms of high CpG content promoters show slightly damped amplitudes (Figure S7C). These two waves of transcription preceded biphasic mRNA accumulation, albeit not as sharp, centered on ZT15 and ZT1 (Figure 5A, mRNA). Both the delay and less sharp bimodality are expected from mRNA processing and turnover times, the latter ranging from minutes to days with a mean of a few hours in mammalian cells [45],[46]. At the measured resolution, the Pol II promoter and gene body rhythms were, on average, in sync (Figure 5B). Interestingly, the phases of H3K4me3 were delayed, on average by 1.3 h, compared to Pol II. This is consistent with the 1 h delay reported for the *Dbp* gene [9], indicating that while H3K4me3 levels stay high at the troughs of diurnal transcription (Figure 4D,E), the maximum H3K4me3 levels slightly lag maximal transcription. Moreover, the peak times of H3K36me3 and mRNA maximum accumulation were shifted by an average of about 3 h relative to Pol II occupancy (Figure 5B,C). Both the overall levels of Pol II, H3K4me3, and H3K36me3 (Figure S7D) and phase relationships (Figure S7E) did not depend on the phase of transcription. Moreover, the identified average phase relationships and delays were unchanged with the extended gene selection (Figure S8).

### Temporal Relationships of Pol II Occupancy and mRNA Accumulation Suggest Widespread Posttranscriptional Control

**Figure 6:**
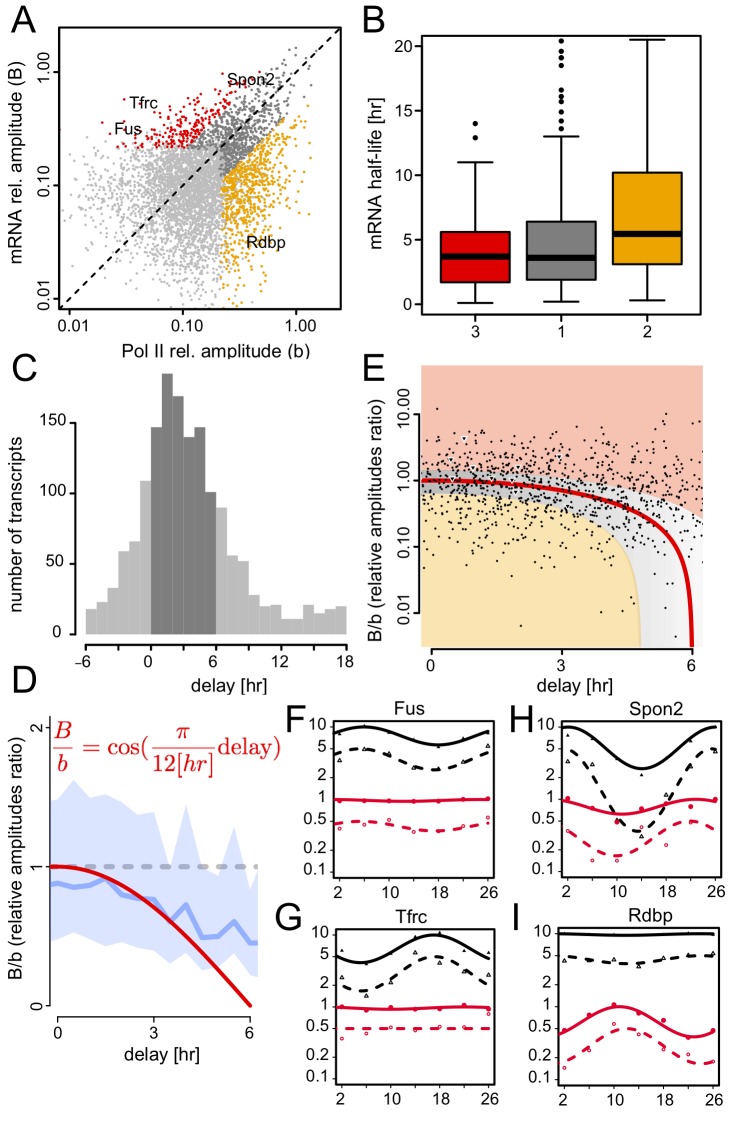
Amplitude and phase relationships between Pol II signals and mRNA accumulation identify posttranscriptional regulation in mRNA accumulation. (A) Relative amplitudes (maximum minus minimum, divided by twice the mean after background subtraction; see Materials and Methods) of oscillations in Pol II promoter signals and mRNA accumulation identify rhythmic mRNAs with relative amplitudes comparable to that of transcription (class 1, gray, 675 genes), long-lived transcripts with damped mRNA rhythms (class 2, orange, 668 genes), and mRNAs where posttranscriptional regulation increases rhythmic amplitude (class 3, red, 217 genes). Light gray genes are all genes that cycle robustly in either Pol II or mRNA accumulation (3,446 genes). *Fus*, *Tfrc*, and *Spon2* are representative of class 3 and *Rdbp* of class 2 (see panels F–I for qRT-PCR validations). The few values larger than 1 are due to low signals when background subtraction makes trough values negative. (B) Half-lives of the three classes taken from NIH-3T3 fibroblasts [46] show a significant difference (TukeyHSD *p* value <10^−6^ for class 2 versus class 1, and class 2 versus class 3). (C) Delays in peak mRNA accumulation versus peak Pol II promoter loading for the union of class 1, class 2, and class 3 genes. The dark gray region delimits the range predicted for a model with constant half-lives (0 h delay for very short-lived up to 6 h for very long-lived transcripts). (D–E) For the same genes the ratio of relative amplitudes (*B* = relative amplitude of Pol II, *b* = relative amplitude of mRNA) is plotted against the phase delay, together with the prediction for a constant half-life (red line). The trend (in D, median is the blue line and 25% and 75% percentiles are shown as light blue shading) shows that the ratio is centered on one at short delays and decreases for larger delays. The scatter plot (E) highlights genes for which transcript accumulation is explained by a constant half-life (dark gray area represents short and light gray long half-lives), and genes where nonconstant half-lives either suppress (light orange) or enhance (light red) amplitudes in mRNA accumulation. Triangles show core circadian clock genes. (F–I) Transcription and mRNA accumulation for representative genes. Comparison of (i) mRNA levels as measured by gene expression arrays, (ii) promoter Pol II occupancy as measured by ChIP-Seq, and (iii) pre-mRNA and (iv) mRNA levels as measured by qRT-PCR with intronic and exonic probes, respectively. Symbols and lines indicate measurements and cosine fits, respectively. Open symbols and dashed lines show qRT-PCR data (cDNA from *n* = 4 animals where pooled) with circles for the pre-mRNA and triangles for the mRNA. Continuous lines and filled symbols represent Pol II ChIP-seq (circles) and mRNA Affymetrix data (triangles). Each temporal profile has been scaled to an arbitrary mean for visualization. Pre-mRNA levels closely follow Pol II promoter occupancy, as expected (given the short half-lives of pre-mRNAs). *Fus* and *Spon2* (F and H) show higher amplitude in mRNA compared to transcription; *Tfrc* (G) is transcribed at similar rates around the clock but shows rhythmic mRNA accumulation; *Rdbp* (I) shows rhythmic transcription but dampened mRNA accumulation.

To establish whether rhythmically accumulating mRNA can be explained from rhythmic transcription, we quantitatively compared phases and amplitudes of Pol II occupancy with mRNA levels. While the relative importance of transcriptional amplitudes and mRNA stability leads to a continuum of mRNA phases and amplitudes (cf., Kinetic model for mRNA accumulation, Materials and Methods), we chose to discuss qualitatively different outcomes by distinguishing three classes of genes (Table S4; Materials and Methods). Class 1 genes (*n* = 675) were rhythmic with regard to both transcription and mRNA accumulation. Class 2 genes (*n* = 668) were transcribed rhythmically, but their RNAs accumulated at almost constant levels. Finally, class 3 genes (*n* = 217) were constitutively transcribed, but their RNAs accumulated in a rhythmic manner (Figure 6A). Cases of class 2 genes have been described previously and include the serum albumin (*Alb*) gene [47], whose mature transcripts are relatively stable. Thus, mRNAs issued from class 2 genes are probably longer lived than mRNAs specified by class 1 genes, consistent with mRNA half-life measurements in mouse fibroblasts (Figure 6B) [46]. The rhythmic accumulation of mRNAs produced by class 3 genes is probably driven by posttranscriptional mechanisms, such as oscillating processing efficiencies and degradation rates (Figure 6B). The comparison of class 1 and class 3 genes—that is, those with rhythmic mRNAs—overlapped highly with previous transcriptome analyses (Figure S9) [41],[43]. Gene Ontology analysis indicated that class 1 genes are strongly enriched in rhythmic hepatic function (e.g., involving carbohydrate and lipid metabolism). On the other hand, class 2 and class 3 genes were not associated with these canonical diurnal functions, showing no obvious functional themes (Tables S5, S6, S7). A kinetic model that considers a cosine-shaped synthesis rate with a 24-h period and constant degradation predicted that the mRNA accumulation can be delayed maximally by 6 h for very long-lived transcripts, which is consistent for the majority of the transcripts (Figure 6C). Delays outside this range must thus reflect the cyclic control of posttranscriptional events. A further prediction is that the ratio of relative amplitudes in mRNA accumulation versus transcription follows a cosine function of the delay, which is consistent with the bulk of our data, emphasizing that, on average, our Pol II amplitudes from ChIP-seq are in quantitative relationship with the measured microarray mRNA accumulations (Figure 6D). The study of individual transcripts showed that some class 2 genes (with damped mRNA amplitudes) indeed show accumulation phases that are consistent with constant long-lived transcripts (Figure 6E, light gray region), while others have compressed mRNA amplitudes but only small phase delays, suggesting a nonconstant transcript half-live that buffers rhythmic transcription (Figure 6E, orange region). On the opposite end, temporally varying half-lives may enhance diurnal amplitudes in mRNA accumulation beyond what is expected from transcription (Figure 6E, red region). Examples of the latter case (class 3 genes) were *Fus*, *Tfrc*, and *Spon2*, whose cyclic accumulation was confirmed by a quantitative RT-PCR analysis (Figure 6F–H). In contrast, *Rdbp* (class 2 gene) showed flat mRNA accumulation despite rhythmic transcription (Figure 6I).

## Discussion

Our genome-wide temporal profiling of Pol II occupancy and H3K4me3 and H3K36me3 histone marks in mouse liver provided quantitative insights into the kinetic relationships between Pol II occupancy at promoters and Pol II occupancy in genes bodies, as well as the kinetics of deposition and removal of histone marks.

### Rhythmic Recruitment of Pol II Rather Than Rhythmic Pol II Release Underlies Diurnal Transcription

In mammalian in vitro Pol II transcription systems, in which the rate for de novo assembly of the transcriptional machinery at gene promoters is extremely low [48],[49], the net transcription rate is thus set by Pol II recruitment to the DNA template. This has led to the idea that transcription in vivo is similarly controlled by Pol II recruitment to promoters by specific DNA binding factors. On the other hand, it has long been known that on certain promoters, an important regulation occurs after the recruitment and initiation steps by controlling the transition from a promoter proximal paused state to active elongation [50]–[52]. In fact, recent results suggest that the regulation of this transition determines transcription output in up to one-third of the genes actively transcribed in ES cells [34]. Our finding that the temporal accumulation of Pol II at promoter proximal regions was in phase with Pol II occupancy within gene bodies strongly suggests that the rhythmic recruitment of Pol II to gene promoters is the principal step determining diurnal transcription rhythms in mouse liver. That is, the subsequent transitions, mainly initiation and de-pausing, do not significantly influence those rhythms. This hypothesis was supported by a simplified mathematical model of transcription assuming cyclic variation of either Pol II recruitment or elongation rates (Figure S4). Indeed, the genes displaying a profile consistent with an elongation-limited scenario (i.e., showing either profile with low amplitude in the Pol II levels at promoters compared to those in gene bodies or anticorrelated Pol II profiles at promoters and in gene bodies) were a minority of genes expressed at background levels (Figure S3G). This is in contrast with the situation in proliferating ES cells [34],[53], where c-Myc controls a large fraction of genes by activating transcription elongation, and probably reflects a different mode by which transcription factors control daily transcription rhythms in a differentiated tissue. It will be interesting to compare our total Pol II occupancy (as detected by our ChIPs with antibodies directed against the second subunit of Pol II) with occupancies of Pol II phosphorylated either on Ser 5, or on both Ser 5 and 2, of the CTD. Of note, a recent study performed under constant darkness conditions (circadian CT times) reported that, unlike our bimodal transcription phases (Figure 5), promoter proximal Ser5-phosphorylated Pol II ChIP signals show a single dominating peak phased around CT1, while hypophosphorylated Pol II sharply peaked at the different CT16 phase [20]. Further experiments, including localization of Ser2 phosphorylated Pol II, will be necessary to understand the dynamic progression of Pol II states during ZT conditions and may reveal differences between ZT and CT sampling. Such differences, as well as differential experimental methodologies and reagents, may also explain why our analysis did not reveal the antiphasic accumulation of Pol II downstream the PASs of the negative core clock regulators Per1 and Cry2 (Figures 4C and S10), as reported in [19].

### Dynamic Changes in Histone Marks Indicate Daily Remodeling of Epigenetic Landscapes

Our temporal analysis of Pol II occupancy and H3K4me3 and H3K36me3 histone marks in mouse liver revealed that the maxima in Pol II occupancy in promoters (or gene bodies) preceded peaks of H3K4me3 by 1 h, H3K36me3 by 3 h, and mRNA accumulation by 3 h. While this was unknown for the H3K36me3 mark, a similar finding for H3K4me3 was previously reported for the *Dbp* gene [9]. Such a lag would be consistent with the mechanism of transcription-activation-linked H3K4 trimethylation in yeast, where the responsible Set1 histone methyltransferase is recruited through binding to the Pol II C-terminal domain (CTD) phosphorylated on serine 5 (i.e., after Pol II recruitment to the DNA) [54]. In mammalian cells, however, the recruitment of the corresponding H3K4 methyltransferase (MLL1) occurs at least in part through binding to transcription factors [55]–[57] and indeed through the circadian CLOCK-BMAL1 complex [17], conceivably before Pol II recruitment. On the other hand, H3K4 trimethylation has been shown to be dependent on H2BK120 ubiquitination, itself dependent on assembly of the basal transcription machinery and transcription [58],[59], again suggesting that Pol II might need to be present for trimethylation to occur. The delay in peak accumulation may also reflect a relatively low clearance rate of H3K4me3 as observed in yeast [54]. The H3K36me3 mark accumulated toward the end of genes [24], consistent with the H3K36 methylase associating with the Pol II CTD phosphorylated on serine 2, the phosphorylation mark of elongating polymerase [60]–[64]. Both H3K4me3 and H3K36me3 levels oscillated with shallower daily amplitudes compared to that of transcription, and H3K36me3 was even shallower than H3K4me3. The kinetics of maximum accumulation of the mark depend on both the kinetics of addition and removal from histone H3, but although H3K36 demethylases have been identified, the mechanisms regulating the H3K36me3 mark turnover are not well understood [29],[65]–[67]. Our findings may reflect relatively long half-lives of H3K36me3 marks. Compared to recent data sampled in CT conditions reporting genome-wide, sharply peaked H3K4me3 rhythms at CT18 and at CT20 for H3K36me3 [20], our ZT analysis indicates that the phases for these marks are more continuously spread throughout the diurnal ZT cycle (Figure 5).

### Posttranscriptional Regulation Significantly Contributes to Rhythmic Gene Accumulation

The comparison between mRNA accumulation and Pol II occupation indicated that posttranscriptional mechanisms could strongly contribute to rhythmic diurnal gene expression. Thus, for class 2 genes, Pol II occupancy was cyclic, whereas mRNA accumulation was nearly flat. This would be expected for long-lived transcripts whose syntheses were diurnally rhythmic or, alternatively, in situations where a temporally varying mRNA degradation rate worked against transcription. For class 3 genes, mRNA abundance oscillated in spite of virtually constant polymerase occupancy, suggesting that cyclic pre-mRNA processing efficiency or mRNA decay was subject to diurnal regulation. Consistent with this notion, a rhythmic regulation of Drosophila *per* mRNA decay has previously been shown to contribute to the high amplitude accumulation of Drosophila *per* mRNAs [68]. We should emphasize that our proposed class sizes and class membership may contain significant false positives and negatives owing to our low sampling rate. Nevertheless, the current definitions are rather consistent with the literature (i.e., class 1 contains most core circadian clock genes, class 2 genes contains several long-lived genes such as Albumin, and we validated several class 3 genes by qPCR). Also we were pleased that Pol II and mRNA amplitude were in quantitative relationship, indicating that there is no systematic bias when assessing diurnal rhythms by either method. While denser sampling might be applied in the future to increase statistical confidence, new methods to analyze multiple rhythmic data types will also be required.

### Transcriptional Regulation of Diurnal Protein Accumulation

We have limited our analysis to transcription and mRNA accumulation and can thus not address to what extent the proteins issued by the investigated genes also accumulate in a cyclic fashion. However, the same arguments we have made about RNA synthesis and accumulation also apply for protein synthesis and accumulation. Thus, mRNAs with accumulation rhythms can only produce strongly oscillating proteins if these proteins are short-lived. For example, *Bmal1* mRNA accumulates with an about 30-fold amplitude, while due to a long half-life, BMAL1 protein levels only fluctuate about 3-fold in abundance [27]. Surprisingly, a large fraction of liver proteins found to be cyclic in liver appear to be encoded by nonoscillating mRNAs [69]. Hence, diurnally controlled translation and/or decay rates must account for the rhythmic accumulation of these proteins. We considered a scenario in which diurnal transcription might result in rhythmic translation of stably accumulating mRNAs. Since polyA length is an important mRNA element in the regulation of translation efficiency [70], it is conceivable that newly synthesized mRNA with longer poly(A) tails are more efficiently translated than older mRNAs. Hence, protein synthesis would follow transcription rather than mRNA accumulation. At least for two genes, *Gstm1* and *Bhmt*, which encode relatively stable mRNAs but oscillating proteins, the phases of Pol II occupancy and protein accumulation would be compatible with such a mechanism. Conceivably, the proposed scenario could apply to additional proteins that, due to their low abundance, have escaped detection in reference [69]. Further work will be required to examine whether cyclic transcription rates can lead to rhythmic translation rates in spite of compressed total mRNA accumulation profiles and, more generally, to study the contributions of cyclic protein degradation rates to the diurnally rhythmic proteome.

## Materials and Methods

### Animals

C57/BL6 male, 12–14-wk-old (at time of sacrifice), mice were housed in a 12 h light/12 h dark (LD) regimen for 2 wk with water and food available *ad libitum*. They were then phase-entrained to a 12 h/12 h LD regimen with water *ad libitum* but food access between ZT12 and ZT24 for 7 d (ZT, Zeitgeber time; ZT0 is defined as the time when the lights are turned on and ZT12 as the time when lights are turned off). At each ZT2, ZT06, ZT10, ZT14, ZT18, ZT22, and ZT26, five mice were anesthetized with isoflurane and decapitated. The livers were perfused with 2 ml of PBS through the spleen and immediately collected. A small piece of liver tissue (approx. 100 mg) was snap-frozen in liquid nitrogen and kept at −80°C for RNA extraction. The remaining liver tissue was immediately homogenized in PBS containing 1% formaldehyde for chromatin preparation. All animal care and handling was performed according to the State of Geneva's law for animal protection.

### Chromatin Immunoprecipitation (ChIP)

Perfused livers were processed for chromatin preparation as described in [9]. The chromatin samples from the five mice were then pooled, frozen in liquid nitrogen, and stored at −80°C. For the ChIP experiments, the following antibodies were used: anti-RPB2 (Santa Cruz Biotechnology, sc-673-18), anti-H3K4me3 (Abcam, ab8580), and anti-H3K36me3 (Abcam, ab9050). To determine the optimal amounts of each antibody, we performed pilot ChIP assays and determined the enrichment for a set of promoters by real-time qPCR according to [9] (not shown).

A total of 1 ml of each chromatin suspension (containing about 60 µg of DNA) was incubated with 10 µg of anti-RPB2, 1.5 µg of anti-H3K36me3, or 1.5 µg of anti-H3K4me3 in buffer A (20 mM Tris/HCl (pH 7.5), 150 mM NaCl, 2 mM EDTA) overnight at 4°C on a rotating wheel. Ten µl of protein A bead suspension (25% slurry in buffer A), pre-blocked with 10 µg/ml of salmon sperm DNA and BSA at 4°C overnight, was then added and the incubation was continued for 1 h at room temperature on a rotating wheel. The beads were then washed with dialysis buffer and ChIP wash buffer as described [71]. Protein–DNA complexes were eluted from the beads, de-cross-linked, and treated with RNase A and, subsequently, with proteinase K, as described [71]. The DNA concentration was determined by fluorometry on the Qubit system (Invitrogen). A total of 10–12 ng DNA were used for the preparation of the library. Libraries for ultra-high throughput sequencing were prepared with the ChIP-Seq DNA sample kit (Illumina) as recommended by the manufacturer.

### RNA Isolation and Analysis

About 100 mg of snap-frozen liver tissue were used for RNA preparation with the TRIzol reagent (Invitrogen) according to the manufacturer's instructions and purified with a miRNeasy Mini Kit (Qiagen). For each time point, 500 ng of total RNA from each of the five mouse livers were pooled and analyzed on Mouse Gene 1.0ST arrays according to the manufacturer's instructions (Affymetrix). All statistical analyses were performed with the statistical language R and various Bioconductor packages (http://www.Bioconductor.org). Normalized expression signals were calculated from Affymetrix CEL files using RMA normalization method [72].

### Quantitative Reverse Transcriptase-PCR Analysis

cDNA was synthesized from 2 µg of liver whole-cell RNA using random hexamers and Superscript II reverse transcriptase (RT) (Invitrogen) following the supplier's instructions. Five percent of this cDNA was PCR amplified (7900HT Sequence Detection Systems, Applied Biosystems) with the Sybr Green master mix (Applied Biosystems), and raw threshold-cycle (Ct) values were calculated with SDS 2.0 software (Applied Biosystems). Mean values were calculated from triplicate PCR assays for each sample and normalized to those obtained for *Cyclophillin* and *GusB* transcripts, which accumulate at invariable levels throughout the day and thus served as internal controls [73]. PCR primers used are listed in Table S5.

### ChIP-Seq Data Analysis and Read Mapping

At each time point, DNA sequenced reads were mapped to the mouse genome (*Mus musculus* NCBI m37 genome assembly (mm9; July 2007)) using Bowtie [74] with three mismatches and at most five hits allowed on the genome. When computing genomic read densities, each alignment contributed 1/(total number of hits) to the local density. If several reads coming from the same library mapped at the same genomic position and on the same strand (redundant tags), we considered this as a PCR artifact and kept only one read for the rest of the analysis. The total numbers of reads per time point are given in Table S1.

### Averaged Density Profiles (Figure 2 and S2)

A set of 11,217 (Table S2) transcription units was selected for analysis as follows: for each Ensembl gene, the most upstream TSS and the most downstream PAS were selected. To ensure that spatial profiles collected around TSS and PAS were not distorted by signals from other genes, we selected TSSs and PASs removed by at least 1.5 kb from documented TSSs and PASs from any other gene. We further limited our analysis to genes for which we could unambiguously assign an Affymetrix Mouse Gene 1.0 ST microarray probe set, to permit stratification of our spatial profiles by expression levels.

### Strand Shifting

Tags mapped on the + and − strands were shifted to account for the length of the inserts in the libraries and then merged. The optimal shifts were computed for each sample using cross-correlation analysis on the sets of TSS or PAS segments selected as described above. The shift values applied to both strands were in the range of 30–50 bp.

### Normalization and Quantification of ChIP-Seq Data

To normalize for differences in sequencing depth among the time points, the number of tags per genomic position in each ChIP-Seq library was first rescaled by the total number of mapped tags. Annotated Ensembl genes were used to quantify the Pol II signals in promoters (window of ±1 kb around annotated TSSs), gene bodies (windows from +1 kb after the TSS to the PAS excluding any ±1 kb regions around internal promoters) and immediately downstream of the PASs (PAS to +1 kb). The H3K4me3 signals were quantified in a region extending from ±1 kb around TSSs. As the H3K36me3 signal was found mostly in the gene bodies towards the end of the genes, the last 40% of gene bodies were used for quantification. For each of these features, the signal densities were then quantile normalized across time points (data in Table S3). For the genomic profiles in Figures 1 and S1, the densities at each genomic position were normalized according to those quantile normalizations. For optimal visualization, spatial smoothing using ±1 kb running windows was then applied.

### Cosine Fits

We used the function *x*(*t*) = *A*_0_+*A*_24_cos(2*π*/(24 h) * (*t*−*t*_max_)) to perform least squared fitting of temporal profiles. *A*_0_ is the mean signal, *A*_24_ the amplitude of the diurnal oscillation, and *t*_max_ the peak time. Such fits are conveniently done using Fourier series.

### Selection of Transcripts for Each Gene

In Figures 3–6, one transcription unit per gene was selected that corresponded to the most highly occupied TSS and PAS for each gene (the selected transcripts are listed in Table S4, column 3). Specifically we considered the highest geometric mean of Pol II at promoters, Pol II in the first kb after the PAS (Figures 2B and 3B), and H3K4me3 at promoters (for each mark the average over time-points was taken).

### Rhythmicity Analysis and Gene Selections

The 24-h spectral power and phase were computed by using established methods [11], and the *p* value associated with 24 h rhythmic profiles was computed using the Fisher test for one specific period [75]. For Figure 5, we used the Fisher combined probability test on the Pol II promoter and body signals, H3K4me3, H3K36me3, and mRNA to select transcripts. Specifically, we computed the Fisher rhythmicity *p* values (see above) for each of those *k* = 5 marks and combined them using the statistics 
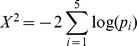
, which assumes a Chi-squared distribution with 2*k* degree of freedom [76]. The resulting combined *p* value was used to estimate False Discovery Rates (FDR) via the linear step-up method [77]. The number of genes selected in function of FDR is shown in Figure S6E, and cutoffs of FDR = 0.3 and FDR = 0.5 were used in Figure 5 and Figure S8, respectively. For Figure 6, genes were selected that cycled either in Pol II occupancy or in mRNA accumulation. The first criterion was that the two signals should be measured above background—that is, transcripts were requested to have mean Pol II loading above 1.9 in Pol II signals (background was estimated at 0.94) and mean mRNA expression (in natural scale) above 85 units in mRNA expression (background was at 43). Secondly the temporal profiles of either Pol II or mRNA had to exhibit high-amplitude rhythmic patterns (fold change greater than 1.5 and cycling *p* value <0.25; for Pol II the *p* value was obtained from the promoter and gene body cycling *p* values, combined with the Fisher Chi-squared test with *k* = 2). This set of 1,560 genes was further split into three classes according to the relative amplitudes *B* = *A_24_*/*A*_0_ computed from the cosine fits. We defined class 3 genes (*n* = 217) as those with *B*(mRNA)/*B*(Pol II)>2; class 2 (*n* = 668) with *B*(mRNA)/*B*(Pol II)<0.5; and class 1 genes (*n* = 675) as the remaining set. All selections are provided in Table S4. Temporal profiles for the three gene classes are viewable at http://cyclix.vital-it.ch.

### Kinetic Model for mRNA Accumulation

To study amplitude and phase relationships between transcription and mRNA accumulation, we used a simplified kinetic model for the accumulation of the mRNA *m*(*t*):
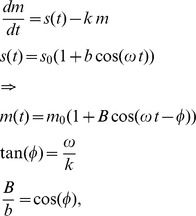
in which the synthesis function *s*(*t*) is taken as a cosine function with frequency *ω* = 2*π*/24*h* ^−1^ mean *s*_0_, and relative amplitude *b*. *k* is a constant mRNA degradation rate. This linear model predicts that the mRNA accumulate as a cosine function with mean *m*_0_, relative amplitude *B*, and delay *φ* with respect to synthesis. The tangent of delay is given by the ratio of the frequency to the degradation rate, which constrains the delay between 0 and 6 h (the former for short and the latter for very long half-lives). Moreover the ratio of relative amplitudes is given by the cosine of the phase delay.

### Gene Ontology Analysis

The analysis was performed using GOstats_2.22.0 and the following databases org.Mm.eg.db_2.7.1, KEGG.db_2.7.1, GO.db_2.7.1. In Tables S5, S6, S7, S8, we provide one summary with categories satisfying FDR<0.001 and one sheet for each classes where the GO categories were reported if FDR<0.01.

### Data Availability

Illumina sequencing data for the ChIP-seq and mRNA are available at GEO as the super series GSE35790. Additional processed data and visualization tools are provided at http://cyclix.vital-it.ch.

## Supporting Information

Figure S1 Pol II, H3K4me3, and H3K36me3 profiles measured around the clock. (A) The density profiles of Pol II (red), H3K4me3 (green), and H3K36me3 (blue) are indicated for the *Per1* gene, which spans 11 kb on chromosome 11, with the thin lines above the profiles indicating the position-specific temporal maxima. The gene structure (RefSeq transcripts) is shown below the panel. The *Per1* gene has two alternative TSSs, both with Pol II and H3K4me3 peaks, and both promoters are thus likely active. Maximal Pol II density in gene body is at ZT10. (B) As in (A) but for the constitutively expressed *Tbp* gene, which spans 17.5 kb on chromosome 17. (C) *Per 1* gene, temporal profiles of the quantifications of the different signals. (D) *Tbp* gene, temporal profiles of the quantifications of the different signals.
(TIFF)

Figure S2 Spatial H3K4me3 and H3K36me3 profiles around transcription start sites (TSSs) and polyadenylation sites (PASs) stratified according to mRNA expression levels and CpG content at promoter. (A) Average H3K4me3 signals per transcription unit split by quartile, based on the total mRNA expression level measured by microarray hybridization. Each quartile is represented by a distinct color shading from light (lowest quartile) to dark (upper quartile). (B) As in (A) but around polyadenylation sites (PASs). (C–D) As in (A) and (B) but for the average H3K36me3 signal. (E–F) Average Pol II signals per transcription unit split by quartile, based on the CpG content in windows spanning [−400, +100] around the TSS. Each quartile is represented by a distinct color shading from light (lowest quartile) to dark (upper quartile). (G–H) Input signals at the TSS and PAS.
(TIFF)

Figure S3 Temporal relationship of Pol II promoter loadings and gene body density. (A) H3K36me3 signal in the most 3′-proximal 40% of gene bodies versus Pol II promoter occupancy at ZT2. There is a strong correlation between high Pol II loading at the promoter and high levels of H3K36me3 in the most 3′-proximal 40% of gene bodies. (B) mRNA expression versus Pol II promoter occupancy at ZT2 shows two distinct populations. (C) Pol II occupancies at promoters and in gene bodies vary in synchrony in a genome-wide fashion. Each gene is represented by a line indicating the orientation and the amplitude of changes during a diurnal cycle. Orientation is indicated by the color (key in upper right corner). (D–G) The genes in (A) were separated into groups according to orientation, from genes showing the largest variation in Pol II occupancy within gene bodies (lines with near vertical orientation in D) to genes showing the largest variation within promoter regions (lines with near horizontal orientation in F). Panel G shows genes for which gene body occupancy decreased as promoter region occupancy increased. Most highly expressed genes show patterns of concomitant changes in both promoter and body occupancy (panels D and E). (H) Idem as (C) but for the PAS1K signals against the promoter Pol II signals.
(TIFF)

Figure S4 A model predicts temporal patterns for recruitment- or initiation-regulated rhythmic transcription. (A) Temporal variation of Pol II occupancy at promoter proximal positions (*x*,*y*,*z*) and gene bodies (*w*) as predicted by a simplified model of transcription. The model describes the (reversible) recruitment of polymerases to promoters, the (irreversible) transition to the open complex, the (irreversible) promoter escape, and the (irreversible) transition from a pausing to an elongating state, after which the polymerases travel to the end of the transcript. We investigate the two scenarios in which either the recruitment or the elongation rates are subject to cyclic circadian variation. (B) The mathematical model of transcription and its parameters: *k_f_* (forward recruitment rate), *k_b_* (backward recruitment rate), *k_o_* (isomerization rate), *k_i_* (promoter escape rate), *k_d_* (de-pausing rate), *v* (elongation rate). (C) Simulated temporal variation of occupancy in promoter proximal positions (*x*,*y*,*z*) and gene body (*w*) when the recruitment rate is varied in a circadian manner. (D) Idem when the de-pausing rate is varied in a circadian manner. The numerical values used in the simulation are *k_f_* = 1/min; *k_b_* = 0; *k_o_* = 0.1/s; *k_i_* = 1/min; *k_d_* = 1/min; *v* = 1/(6 s).
(TIFF)

Figure S5 Temporal relationship of H3K4me3 and H3K36me3 marks with Pol II promoter occupancy. (A and C) Each gene is represented by a line indicating the average orientation and amplitude of changes during a diurnal cycle. Orientation is also indicated by the color (key in upper right corner). (B and D) Binned representation of (A) and (C), respectively. Panel B is identical to Figure 4F and reproduced here for comparison.
(TIFF)

Figure S6 Comparison of gene selection with the Hughes et al. 2009 [41] and Hughes et al. 2012 [43] gene sets. (A) Venn diagram showing the intersection between our gene set (Figure 5, *n* = 284, red) and the rhythmic transcripts in Hughes et al. 2009 (blue) and Hughes et al. 2012 (green). In all pairwise comparisons, the percentage overlaps refer to the smallest of the two sets. Indicated *p* values for the overlaps are computed using the hypergeometric test. The gene sets and overlaps are given in Table S4. From the Hughes et al. 2009 and 2012 datasets, we only considered genes that were also measured on our arrays. Matching was done using the gene symbol. (B) The overlap is stratified according to decreasing mRNA amplitudes (peak to trough). (C–D) Idem for our less stringent gene set (Figure S7, *n* = 752, red). (E) Number of genes in the set as a function of the q-values (Fisher combined probability test with Benjamini-Hochberg FDR correction).
(TIFF)

Figure S7 Stratification of rhythmically transcribed genes into subgroups. (A) Transcription phases for the 400 strongest BMAL1 sites in Rey et al. 2011 [11] show a maximal phase at ZT8, while the 400 strongest REVERBα sites from Cho et al. 2012 [14] show a peak at ZT21. (B) The rhythmic gene set in Figure S7A split into low and high CpG promoters show similar phase distributions. (C) The rhythmic gene set in Figure S7A split into low and high CpG promoters show that high CpG island promoters have slightly lower amplitudes. (D) Mean Pol2 (red), H3K4me3 (green), and H3k36me3 (green) for genes transcribed in the morning phase (phase interval from ZT3 to ZT9) and evening phase (phase interval from ZT15 to ZT21) show no marked difference. (E) Phase delays between H3k4me3 and H3K36me3 compared to Pol II signals are not correlated with the peak time of Pol II (bins are indicated on the *x*-axis).
(TIFF)

Figure S8 Temporal relationships of Pol II, H3K4me3, H3K36me3 profiles, and mRNA accumulation in mouse liver. Idem as Figure 5 with an extended selection of genes (*n* = 752, *p*<0.018, FDR = 0.5).
(TIFF)

Figure S9 Comparison of class 1 and class 3 genes with the Hughes et al. 2009 [41] and Hughes et al. 2012 [43] gene sets. (A) Venn diagram showing the intersection between the genes of class 1 or 3—that is, transcripts that show diurnal variations of mRNA (Figure 6, *n* = 892, red) and the rhythmic transcripts in Hughes et al. 2009 (blue) and Hughes et al. 2012. In all pairwise comparisons, the percentage overlaps refer to the smallest of the two sets. Indicated *p* values for the overlaps are computed using the hypergeometric test. The gene sets and overlaps are given in Table S4. (B) The overlap is stratified according to decreasing mRNA amplitudes (peak to trough).
(TIFF)

Figure S10 Pol II occupancy profiles at the Per1 and Cry2 genes. (A) Profile of Per1, entire locus. (B) Profile of Per1, region around the PAS. (C) Profile of Cry2, entire locus. (D) Profile of Cry2, region around the PAS. The location of ChIP-qPCR primers used in (Padmanhaban et al., Science 2012 [19]) is indicated below the chromosome coordinates.
(TIFF)

Movie S1 Animated profiles for the Bmal1, Reverbα (Nr1d1), and mPer1 genes for the Pol2 (red) occupancy and H3K4me3 (green) and H3K36me3 (blue) marks. The data were interpolated in time using spline interpolation (every 30 min).
(GIF)

Movie S2 Idem as Movie S1 with only the measured time points (no interpolation).
(GIF)

Table S1 Summary of the number of mapped reads per time point per mark.
(XLS)

Table S2 List of the transcription units selected to define the density profiles.
(XLS)

Table S3 Pol II, H3K4me3, and H3K63me3 quantifications after normalization. The signals for each transcript per time point and per mark are given as described in Materials and Methods.
(TXT)

Table S4 Data and gene lists for Figures 5 and 6. Note that the *A*_0_ values use background-subtracted signals (background values were 21 for mRNA expression and 0.47 for Pol II signals).
(TXT)

Table S5 Gene Ontology analysis for the gene in classes 1–3: biological processes.
(XLS)

Table S6 Gene Ontology analysis for the gene in classes 1–3: molecular function.
(XLS)

Table S7 Gene Ontology analysis for the gene in classes 1–3: cellular component.
(XLS)

Table S8 PCR primers used for qRT-PCR validations.
(XLS)

## Abbreviations

ChIP: chromatin immuno-precipitation
CTD: C-terminal domain
PAS: polyadenylation sites
Pol II: RNA polymerase II
RT: reverse transcriptase
TSS: transcript start sites
ZT: Zeitgeber Time

## References

[1] Bass J, Takahashi JS (2010) Circadian integration of metabolism and energetics. Science 330: 1349–1354. 21127246 10.1126/science.1195027 PMC3756146

[2] Kornmann B, Schaad O, Bujard H, Takahashi J, Schibler U (2007) System-driven and oscillator-dependent circadian transcription in mice with a conditionally active liver clock. PLoS Biol 5: e34 doi:10.1371/journal.pbio.0050034. 17298173 10.1371/journal.pbio.0050034 PMC1783671

[3] Lamia KA, Storch KF, Weitz CJ (2008) Physiological significance of a peripheral tissue circadian clock. Proc Natl Acad Sci U S A 105: 15172–15177. 18779586 10.1073/pnas.0806717105 PMC2532700

[4] Huang W, Ramsey KM, Marcheva B, Bass J (2011) Circadian rhythms, sleep, and metabolism. J Clin Invest 121: 2133–2141. 21633182 10.1172/JCI46043 PMC3104765

[5] Rudic RD, McNamara P, Curtis AM, Boston RC, Panda S, et al (2004) BMAL1 and CLOCK, two essential components of the circadian clock, are involved in glucose homeostasis. PLoS Biol 2: e377 doi: 10.1371/journal.pbio.0020377. 15523558 10.1371/journal.pbio.0020377 PMC524471

[6] Gachon F, Leuenberger N, Claudel T, Gos P, Jouffe C, et al (2011) Proline- and acidic amino acid-rich basic leucine zipper proteins modulate peroxisome proliferator-activated receptor alpha (PPARalpha) activity. Proc Natl Acad Sci U S A 108: 4794–4799. 21383142 10.1073/pnas.1002862108 PMC3064322

[7] Le Martelot G, Claudel T, Gatfield D, Schaad O, Kornmann B, et al (2009) REV-ERBalpha participates in circadian SREBP signaling and bile acid homeostasis. PLoS Biol 7: e1000181 doi:10.1371/journal.pbio.1000181. 19721697 10.1371/journal.pbio.1000181 PMC2726950

[8] Gachon F, Olela FF, Schaad O, Descombes P, Schibler U (2006) The circadian PAR-domain basic leucine zipper transcription factors DBP, TEF, and HLF modulate basal and inducible xenobiotic detoxification. Cell Metab 4: 25–36. 16814730 10.1016/j.cmet.2006.04.015

[9] Ripperger JA, Schibler U (2006) Rhythmic CLOCK-BMAL1 binding to multiple E-box motifs drives circadian Dbp transcription and chromatin transitions. Nat Genet 38: 369–374. 16474407 10.1038/ng1738

[10] Stratmann M, Stadler F, Tamanini F, van der Horst GT, Ripperger JA (2010) Flexible phase adjustment of circadian albumin D site-binding protein (DBP) gene expression by CRYPTOCHROME1. Genes Dev 24: 1317–1328. 20551177 10.1101/gad.578810 PMC2885666

[11] Rey G, Cesbron F, Rougemont J, Reinke H, Brunner M, et al (2011) Genome-wide and phase-specific DNA-binding rhythms of BMAL1 control circadian output functions in mouse liver. PLoS Biol 9: e1000595 doi:10.1371/journal.pbio.1000595. 21364973 10.1371/journal.pbio.1000595 PMC3043000

[12] Feng D, Liu T, Sun Z, Bugge A, Mullican SE, et al (2011) A circadian rhythm orchestrated by histone deacetylase 3 controls hepatic lipid metabolism. Science 331: 1315–1319. 21393543 10.1126/science.1198125 PMC3389392

[13] Bugge A, Feng D, Everett LJ, Briggs ER, Mullican SE, et al (2012) Rev-erbalpha and Rev-erbbeta coordinately protect the circadian clock and normal metabolic function. Genes Dev 26: 657–667. 22474260 10.1101/gad.186858.112 PMC3323877

[14] Cho H, Zhao X, Hatori M, Yu RT, Barish GD, et al (2012) Regulation of circadian behaviour and metabolism by REV-ERB-alpha and REV-ERB-beta. Nature 485: 123–127. 22460952 10.1038/nature11048 PMC3367514

[15] Etchegaray J-P, Lee C, Wade PA, Reppert SM (2003) Rhythmic histone acetylation underlies transcription in the mammalian circadian clock. Nature 421: 177–182. 12483227 10.1038/nature01314

[16] Brown SA, Ripperger J, Kadener S, Fleury-Olela F, Vilbois F, et al (2005) PERIOD1-associated proteins modulate the negative limb of the mammalian circadian oscillator. Science 308: 693–696. 15860628 10.1126/science.1107373

[17] Katada S, Sassone-Corsi P (2010) The histone methyltransferase MLL1 permits the oscillation of circadian gene expression. Nat Struct Mol Biol 17: 1414–1421. 21113167 10.1038/nsmb.1961 PMC6501791

[18] DiTacchio L, Le HD, Vollmers C, Hatori M, Witcher M, et al (2011) Histone lysine demethylase JARID1a activates CLOCK-BMAL1 and influences the circadian clock. Science 333: 1881–1885. 21960634 10.1126/science.1206022 PMC3204309

[19] Padmanabhan K, Robles MS, Westerling T, Weitz CJ (2012) Feedback regulation of transcriptional termination by the mammalian circadian clock PERIOD complex. Science 337: 599–602. 22767893 10.1126/science.1221592

[20] Koike N, Yoo SH, Huang HC, Kumar V, Lee C, et al (2012) Transcriptional architecture and chromatin landscape of the core circadian clock in mammals. Science 10.1126/science.1226339 PMC3694775 22936566

[21] Barski A, Cuddapah S, Cui K, Roh TY, Schones DE, et al (2007) High-resolution profiling of histone methylations in the human genome. Cell 129: 823–837. 17512414 10.1016/j.cell.2007.05.009

[22] Bernstein BE, Kamal M, Lindblad-Toh K, Bekiranov S, Bailey DK, et al (2005) Genomic maps and comparative analysis of histone modifications in human and mouse. Cell 120: 169–181. 15680324 10.1016/j.cell.2005.01.001

[23] Schneider R, Bannister AJ, Myers FA, Thorne AW, Crane-Robinson C, et al (2004) Histone H3 lysine 4 methylation patterns in higher eukaryotic genes. Nat Cell Biol 6: 73–77. 14661024 10.1038/ncb1076

[24] Guenther MG, Levine SS, Boyer LA, Jaenisch R, Young RA (2007) A chromatin landmark and transcription initiation at most promoters in human cells. Cell 130: 77–88. 17632057 10.1016/j.cell.2007.05.042 PMC3200295

[25] Kolasinska-Zwierz P, Down T, Latorre I, Liu T, Liu XS, et al (2009) Differential chromatin marking of introns and expressed exons by H3K36me3. Nat Genet 41: 376–381. 19182803 10.1038/ng.322 PMC2648722

[26] Kojima S, Shingle DL, Green CB (2011) Post-transcriptional control of circadian rhythms. J Cell Sci 124: 311–320. 21242310 10.1242/jcs.065771 PMC3021995

[27] Preitner N, Damiola F, Lopez-Molina L, Zakany J, Duboule D, et al (2002) The orphan nuclear receptor REV-ERBalpha controls circadian transcription within the positive limb of the mammalian circadian oscillator. Cell 110: 251–260. 12150932 10.1016/s0092-8674(02)00825-5

[28] Kim S, Kim H, Fong N, Erickson B, Bentley DL (2011) Pre-mRNA splicing is a determinant of histone H3K36 methylation. Proc Natl Acad Sci U S A 108: 13564–13569. 21807997 10.1073/pnas.1109475108 PMC3158196

[29] de Almeida SF, Grosso AR, Koch F, Fenouil R, Carvalho S, et al (2011) Splicing enhances recruitment of methyltransferase HYPB/Setd2 and methylation of histone H3 Lys36. Nat Struct Mol Biol 18: 977–983. 21792193 10.1038/nsmb.2123

[30] Sims RJ 3rd, Belotserkovskaya R, Reinberg D (2004) Elongation by RNA polymerase II: the short and long of it. Genes Dev 18: 2437–2468. 15489290 10.1101/gad.1235904

[31] Nechaev S, Adelman K (2011) Pol II waiting in the starting gates: regulating the transition from transcription initiation into productive elongation. Biochim Biophys Acta 1809: 34–45. 21081187 10.1016/j.bbagrm.2010.11.001 PMC3021596

[32] Glover-Cutter K, Kim S, Espinosa J, Bentley DL (2008) RNA polymerase II pauses and associates with pre-mRNA processing factors at both ends of genes. Nat Struct Mol Biol 15: 71–78. 18157150 10.1038/nsmb1352 PMC2836588

[33] Sultan M, Schulz MH, Richard H, Magen A, Klingenhoff A, et al (2008) A global view of gene activity and alternative splicing by deep sequencing of the human transcriptome. Science 321: 956–960. 18599741 10.1126/science.1160342

[34] Rahl PB, Lin CY, Seila AC, Flynn RA, McCuine S, et al (2010) c-Myc regulates transcriptional pause release. Cell 141: 432–445. 20434984 10.1016/j.cell.2010.03.030 PMC2864022

[35] Yamashita R, Sathira NP, Kanai A, Tanimoto K, Arauchi T, et al (2011) Genome-wide characterization of transcriptional start sites in humans by integrative transcriptome analysis. Genome Res 21: 775–789. 21372179 10.1101/gr.110254.110 PMC3083095

[36] Brookes E, Pombo A (2009) Modifications of RNA polymerase II are pivotal in regulating gene expression states. EMBO Rep 10: 1213–1219. 19834511 10.1038/embor.2009.221 PMC2775184

[37] Singh J, Padgett RA (2009) Rates of in situ transcription and splicing in large human genes. Nat Struct Mol Biol 16: 1128–1133. 19820712 10.1038/nsmb.1666 PMC2783620

[38] Rosonina E, Kaneko S, Manley JL (2006) Terminating the transcript: breaking up is hard to do. Genes Dev 20: 1050–1056. 16651651 10.1101/gad.1431606

[39] Wang Z, Zang C, Rosenfeld JA, Schones DE, Barski A, et al (2008) Combinatorial patterns of histone acetylations and methylations in the human genome. Nat Genet 40: 897–903. 18552846 10.1038/ng.154 PMC2769248

[40] Ernst J, Kellis M (2010) Discovery and characterization of chromatin states for systematic annotation of the human genome. Nat Biotechnol 28: 817–825. 20657582 10.1038/nbt.1662 PMC2919626

[41] Hughes ME, DiTacchio L, Hayes KR, Vollmers C, Pulivarthy S, et al (2009) Harmonics of circadian gene transcription in mammals. PLoS Genet 5: e1000442 doi:10.1371/journal.pgen.1000442. 19343201 10.1371/journal.pgen.1000442 PMC2654964

[42] Hughes ME, Hogenesch JB, Kornacker K (2010) JTK_CYCLE: an efficient nonparametric algorithm for detecting rhythmic components in genome-scale data sets. J Biol Rhythms 25: 372–380. 20876817 10.1177/0748730410379711 PMC3119870

[43] Hughes ME, Hong HK, Chong JL, Indacochea AA, Lee SS, et al (2012) Brain-specific rescue of clock reveals system-driven transcriptional rhythms in peripheral tissue. PLoS Genet 8: e1002835 doi:10.1371/journal.pgen.1002835. 22844252 10.1371/journal.pgen.1002835 PMC3405989

[44] Ueda HR, Hayashi S, Chen W, Sano M, Machida M, et al (2005) System-level identification of transcriptional circuits underlying mammalian circadian clocks. Nat Genet 37: 187–192. 15665827 10.1038/ng1504

[45] Ross J (1995) mRNA stability in mammalian cells. Microbiol Rev 59: 423–450. 7565413 10.1128/mr.59.3.423-450.1995 PMC239368

[46] Dolken L, Ruzsics Z, Radle B, Friedel CC, Zimmer R, et al (2008) High-resolution gene expression profiling for simultaneous kinetic parameter analysis of RNA synthesis and decay. RNA 14: 1959–1972. 18658122 10.1261/rna.1136108 PMC2525961

[47] Wuarin J, Schibler U (1990) Expression of the liver-enriched transcriptional activator protein DBP follows a stringent circadian rhythm. Cell 63: 1257–1266. 2261643 10.1016/0092-8674(90)90421-a

[48] Hawley DK, Roeder RG (1985) Separation and partial characterization of three functional steps in transcription initiation by human RNA polymerase II. J Biol Chem 260: 8163–8172. 2409080

[49] Hawley DK, Roeder RG (1987) Functional steps in transcription initiation and reinitiation from the major late promoter in a HeLa nuclear extract. J Biol Chem 262: 3452–3461. 2434502

[50] Bentley DL, Groudine M (1986) A block to elongation is largely responsible for decreased transcription of c-myc in differentiated HL60 cells. Nature 321: 702–706. 3520340 10.1038/321702a0

[51] Espinosa JM, Verdun RE, Emerson BM (2003) p53 functions through stress- and promoter-specific recruitment of transcription initiation components before and after DNA damage. Mol Cell 12: 1015–1027. 14580351 10.1016/s1097-2765(03)00359-9

[52] Sawado T, Halow J, Bender MA, Groudine M (2003) The beta-globin locus control region (LCR) functions primarily by enhancing the transition from transcription initiation to elongation. Genes Dev 17: 1009–1018. 12672691 10.1101/gad.1072303 PMC196035

[53] Core LJ, Lis JT (2008) Transcription regulation through promoter-proximal pausing of RNA polymerase II. Science 319: 1791–1792. 18369138 10.1126/science.1150843 PMC2833332

[54] Ng HH, Robert F, Young RA, Struhl K (2003) Targeted recruitment of Set1 histone methylase by elongating Pol II provides a localized mark and memory of recent transcriptional activity. Mol Cell 11: 709–719. 12667453 10.1016/s1097-2765(03)00092-3

[55] Ernst P, Wang J, Huang M, Goodman RH, Korsmeyer SJ (2001) MLL and CREB bind cooperatively to the nuclear coactivator CREB-binding protein. Mol Cell Biol 21: 2249–2258. 11259575 10.1128/MCB.21.7.2249-2258.2001 PMC86859

[56] Yokoyama A, Wang Z, Wysocka J, Sanyal M, Aufiero DJ, et al (2004) Leukemia proto-oncoprotein MLL forms a SET1-like histone methyltransferase complex with menin to regulate Hox gene expression. Mol Cell Biol 24: 5639–5649. 15199122 10.1128/MCB.24.13.5639-5649.2004 PMC480881

[57] Milne TA, Dou Y, Martin ME, Brock HW, Roeder RG, et al (2005) MLL associates specifically with a subset of transcriptionally active target genes. Proc Natl Acad Sci U S A 102: 14765–14770. 16199523 10.1073/pnas.0503630102 PMC1253553

[58] Pavri R, Zhu B, Li G, Trojer P, Mandal S, et al (2006) Histone H2B monoubiquitination functions cooperatively with FACT to regulate elongation by RNA polymerase II. Cell 125: 703–717. 16713563 10.1016/j.cell.2006.04.029

[59] Sims RJ 3rd, Reinberg D (2006) Histone H3 Lys 4 methylation: caught in a bind? Genes Dev 20: 2779–2786. 17043307 10.1101/gad.1468206

[60] Kizer KO, Phatnani HP, Shibata Y, Hall H, Greenleaf AL, et al (2005) A novel domain in Set2 mediates RNA polymerase II interaction and couples histone H3 K36 methylation with transcript elongation. Mol Cell Biol 25: 3305–3316. 15798214 10.1128/MCB.25.8.3305-3316.2005 PMC1069628

[61] Krogan NJ, Kim M, Tong A, Golshani A, Cagney G, et al (2003) Methylation of histone H3 by Set2 in Saccharomyces cerevisiae is linked to transcriptional elongation by RNA polymerase II. Mol Cell Biol 23: 4207–4218. 12773564 10.1128/MCB.23.12.4207-4218.2003 PMC427527

[62] Li J, Moazed D, Gygi SP (2002) Association of the histone methyltransferase Set2 with RNA polymerase II plays a role in transcription elongation. J Biol Chem 277: 49383–49388. 12381723 10.1074/jbc.M209294200

[63] Xiao T, Hall H, Kizer KO, Shibata Y, Hall MC, et al (2003) Phosphorylation of RNA polymerase II CTD regulates H3 methylation in yeast. Genes Dev 17: 654–663. 12629047 10.1101/gad.1055503 PMC196010

[64] Li M, Phatnani HP, Guan Z, Sage H, Greenleaf AL, et al (2005) Solution structure of the Set2-Rpb1 interacting domain of human Set2 and its interaction with the hyperphosphorylated C-terminal domain of Rpb1. Proc Natl Acad Sci U S A 102: 17636–17641. 16314571 10.1073/pnas.0506350102 PMC1308900

[65] Edmunds JW, Mahadevan LC, Clayton AL (2008) Dynamic histone H3 methylation during gene induction: HYPB/Setd2 mediates all H3K36 trimethylation. EMBO J 27: 406–420. 18157086 10.1038/sj.emboj.7601967 PMC2168397

[66] Zee BM, Levin RS, Dimaggio PA, Garcia BA (2010) Global turnover of histone post-translational modifications and variants in human cells. Epigenetics Chromatin 3: 22. 21134274 10.1186/1756-8935-3-22 PMC3004898

[67] Berger SL (2007) The complex language of chromatin regulation during transcription. Nature 447: 407–412. 17522673 10.1038/nature05915

[68] So WV, Rosbash M (1997) Post-transcriptional regulation contributes to Drosophila clock gene mRNA cycling. EMBO J 16: 7146–7155. 9384591 10.1093/emboj/16.23.7146 PMC1170315

[69] Reddy AB, Karp NA, Maywood ES, Sage EA, Deery M, et al (2006) Circadian orchestration of the hepatic proteome. Curr Biol 16: 1107–1115. 16753565 10.1016/j.cub.2006.04.026

[70] Gebauer F, Hentze MW (2004) Molecular mechanisms of translational control. Nat Rev Mol Cell Biol 5: 827–835. 15459663 10.1038/nrm1488 PMC7097087

[71] O'Geen H, Nicolet CM, Blahnik K, Green R, Farnham PJ (2006) Comparison of sample preparation methods for ChIP-chip assays. Biotechniques 41: 577–580. 17140114 10.2144/000112268 PMC2268903

[72] Irizarry RA, Hobbs B, Collin F, Beazer-Barclay YD, Antonellis KJ, et al (2003) Exploration, normalization, and summaries of high density oligonucleotide array probe level data. Biostatistics 4: 249–264. 12925520 10.1093/biostatistics/4.2.249

[73] Vandesompele J, De Preter K, Pattyn F, Poppe B, Van Roy N, et al (2002) Accurate normalization of real-time quantitative RT-PCR data by geometric averaging of multiple internal control genes. Genome Biol 3: RESEARCH0034. 12184808 10.1186/gb-2002-3-7-research0034 PMC126239

[74] Langmead B, Trapnell C, Pop M, Salzberg SL (2009) Ultrafast and memory-efficient alignment of short DNA sequences to the human genome. Genome Biol 10: R25. 19261174 10.1186/gb-2009-10-3-r25 PMC2690996

[75] Fisher RA (1929) Tests of significance in harmonic analysis. Proceedings of the Royal Society of London Series A-Containing Papers of a Mathematical and Physical Character 125: 54–59.

[76] Fisher RA (1925) Statistical methods for research workers. Edinburgh, UK: Oliver and Boyd.

[77] Benjamini Y, Hochberg Y (1995) Controlling the false discovery rate: a practical and powerful approach to multiple testing. Journal of the Royal Statistical Society Series B 57: 289–300.

