# Supplementary figures and images for: Genome-Wide RNA Polymerase II Profiles and RNA Accumulation Reveal Kinetics of Transcription and Associated Epigenetic Changes During Diurnal Cycles
Source: PLoS Biol. 2012 Nov 27;10(11):e1001442. doi: 10.1371/journal.pbio.1001442 (PMC3507959; doi:10.1371/journal.pbio.1001442)

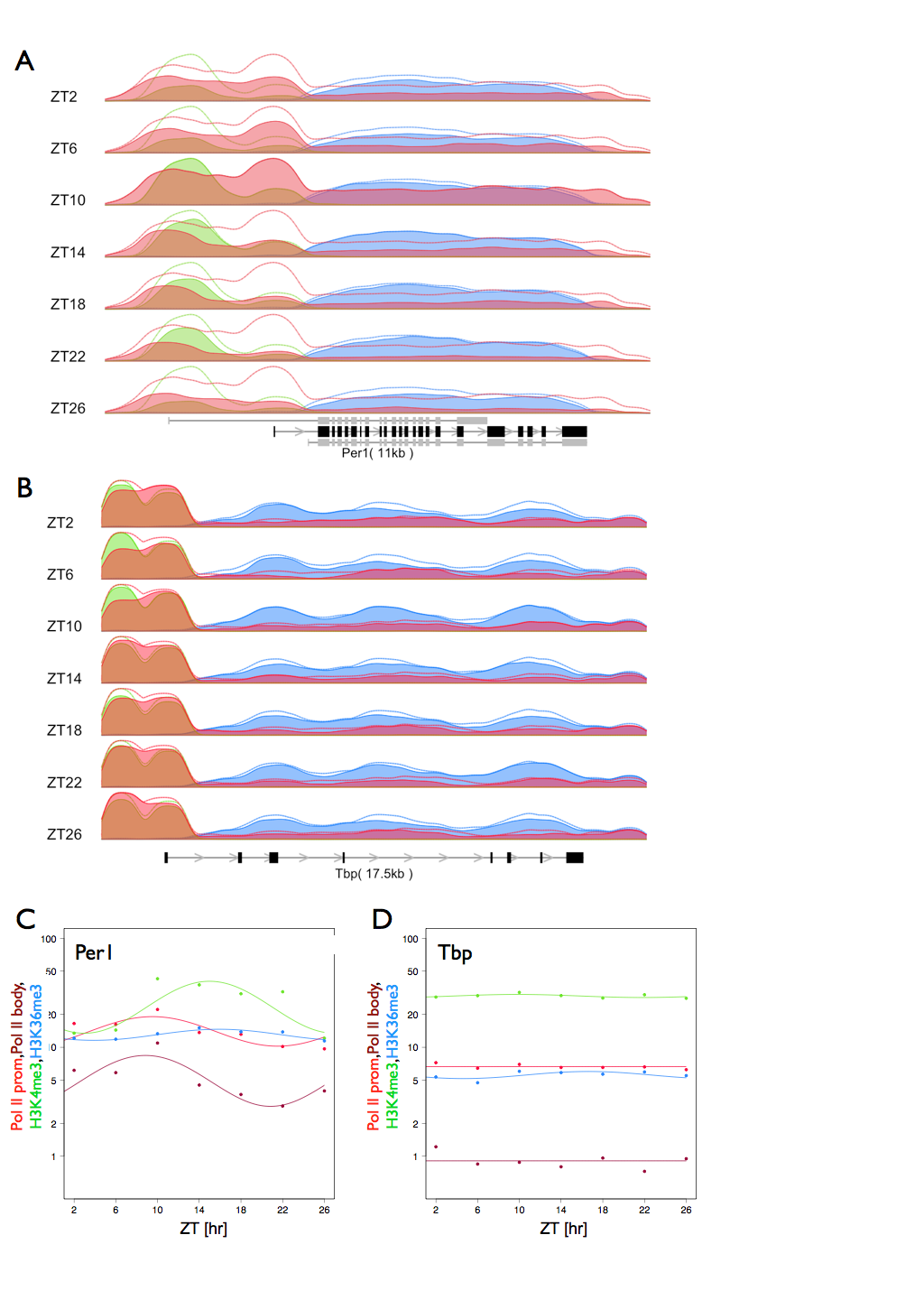

Supplement: Figure S1 — Pol II, H3K4me3, and H3K36me3 profiles measured around the clock. (A) The density profiles of Pol II (red), H3K4me3 (green), and H3K36me3 (blue) are indicated for the Per1 gene, which spans 11 kb on chromosome 11, with the thin lines above the profiles indicating the position-specific temporal maxima. The gene structure (RefSeq transcripts) is shown below the panel. The Per1 gene has two alternative TSSs, both with Pol II and H3K4me3 peaks, and both promoters are thus likely active. Maximal Pol II density in gene body is at ZT10. (B) As in (A) but for the constitutively expressed Tbp gene, which spans 17.5 kb on chromosome 17. (C) Per 1 gene, temporal profiles of the quantifications of the different signals. (D) Tbp gene, temporal profiles of the quantifications of the different signals. (TIFF) [file pbio.1001442.s001.tiff]

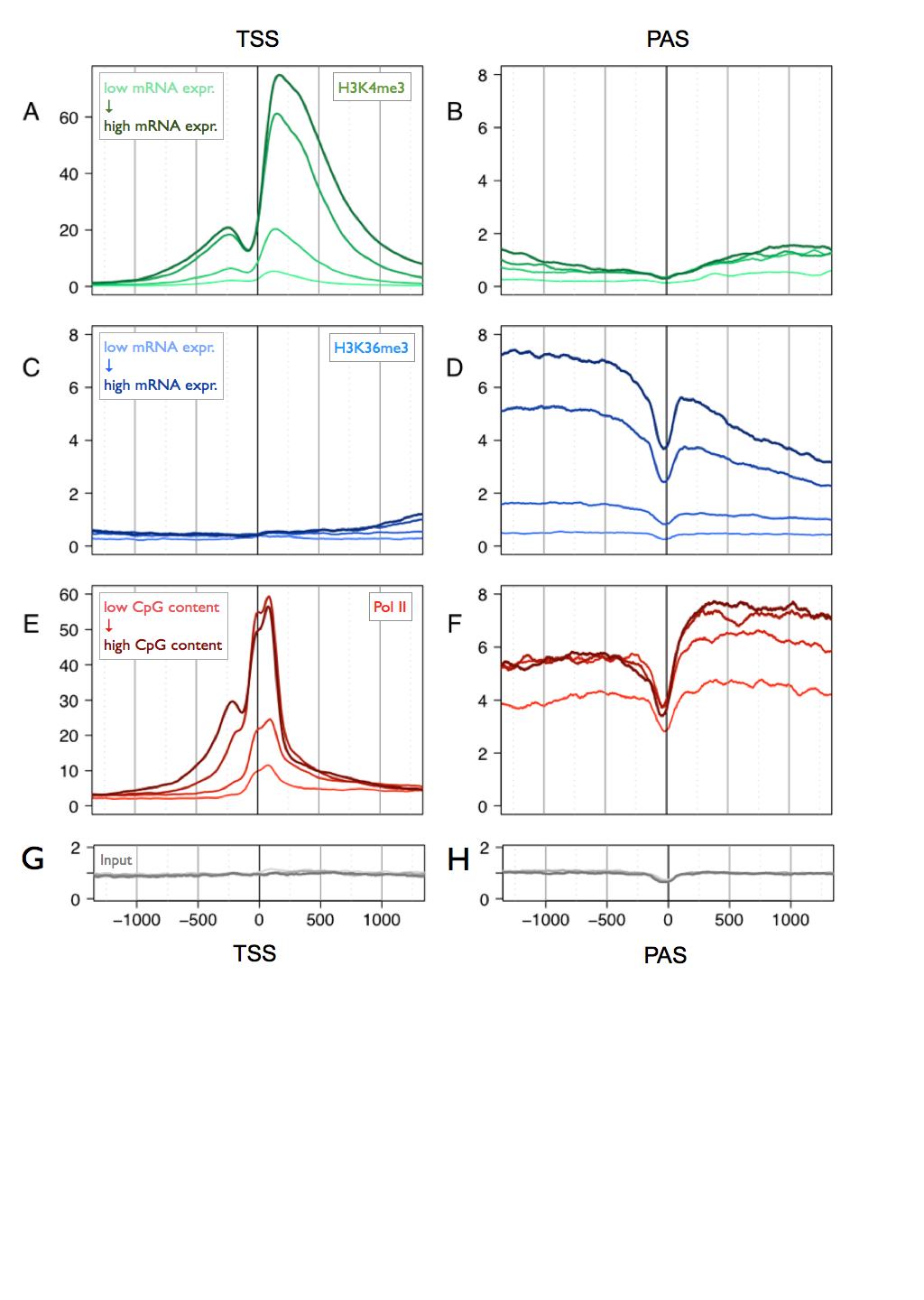

Supplement: Figure S2 — Spatial H3K4me3 and H3K36me3 profiles around transcription start sites (TSSs) and polyadenylation sites (PASs) stratified according to mRNA expression levels and CpG content at promoter. (A) Average H3K4me3 signals per transcription unit split by quartile, based on the total mRNA expression level measured by microarray hybridization. Each quartile is represented by a distinct color shading from light (lowest quartile) to dark (upper quartile). (B) As in (A) but around polyadenylation sites (PASs). (C–D) As in (A) and (B) but for the average H3K36me3 signal. (E–F) Average Pol II signals per transcription unit split by quartile, based on the CpG content in windows spanning [−400, +100] around the TSS. Each quartile is represented by a distinct color shading from light (lowest quartile) to dark (upper quartile). (G–H) Input signals at the TSS and PAS. (TIFF) [file pbio.1001442.s002.tiff]

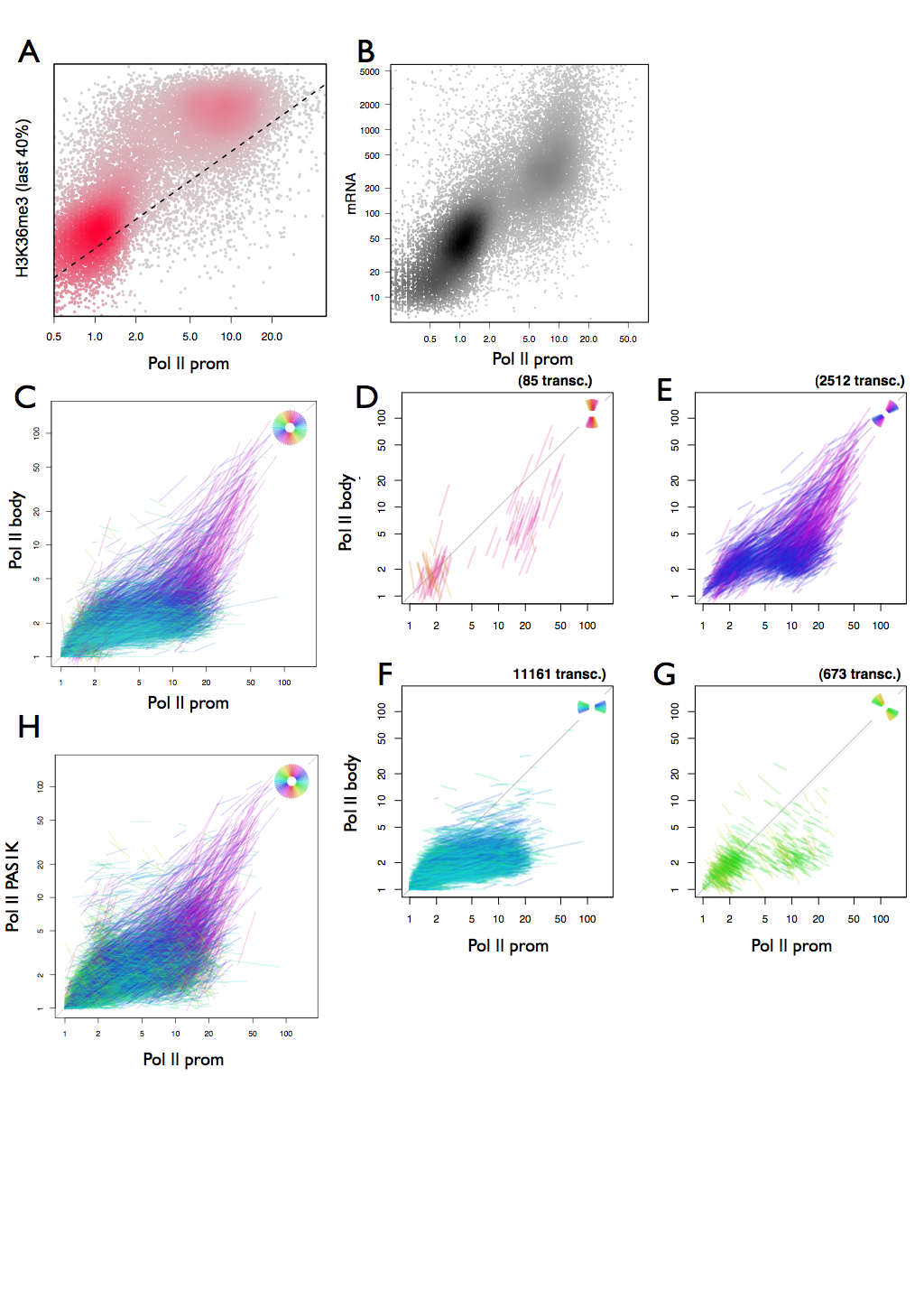

Supplement: Figure S3 — Temporal relationship of Pol II promoter loadings and gene body density. (A) H3K36me3 signal in the most 3′-proximal 40% of gene bodies versus Pol II promoter occupancy at ZT2. There is a strong correlation between high Pol II loading at the promoter and high levels of H3K36me3 in the most 3′-proximal 40% of gene bodies. (B) mRNA expression versus Pol II promoter occupancy at ZT2 shows two distinct populations. (C) Pol II occupancies at promoters and in gene bodies vary in synchrony in a genome-wide fashion. Each gene is represented by a line indicating the orientation and the amplitude of changes during a diurnal cycle. Orientation is indicated by the color (key in upper right corner). (D–G) The genes in (A) were separated into groups according to orientation, from genes showing the largest variation in Pol II occupancy within gene bodies (lines with near vertical orientation in D) to genes showing the largest variation within promoter regions (lines with near horizontal orientation in F). Panel G shows genes for which gene body occupancy decreased as promoter region occupancy increased. Most highly expressed genes show patterns of concomitant changes in both promoter and body occupancy (panels D and E). (H) Idem as (C) but for the PAS1K signals against the promoter Pol II signals. (TIFF) [file pbio.1001442.s003.tiff]

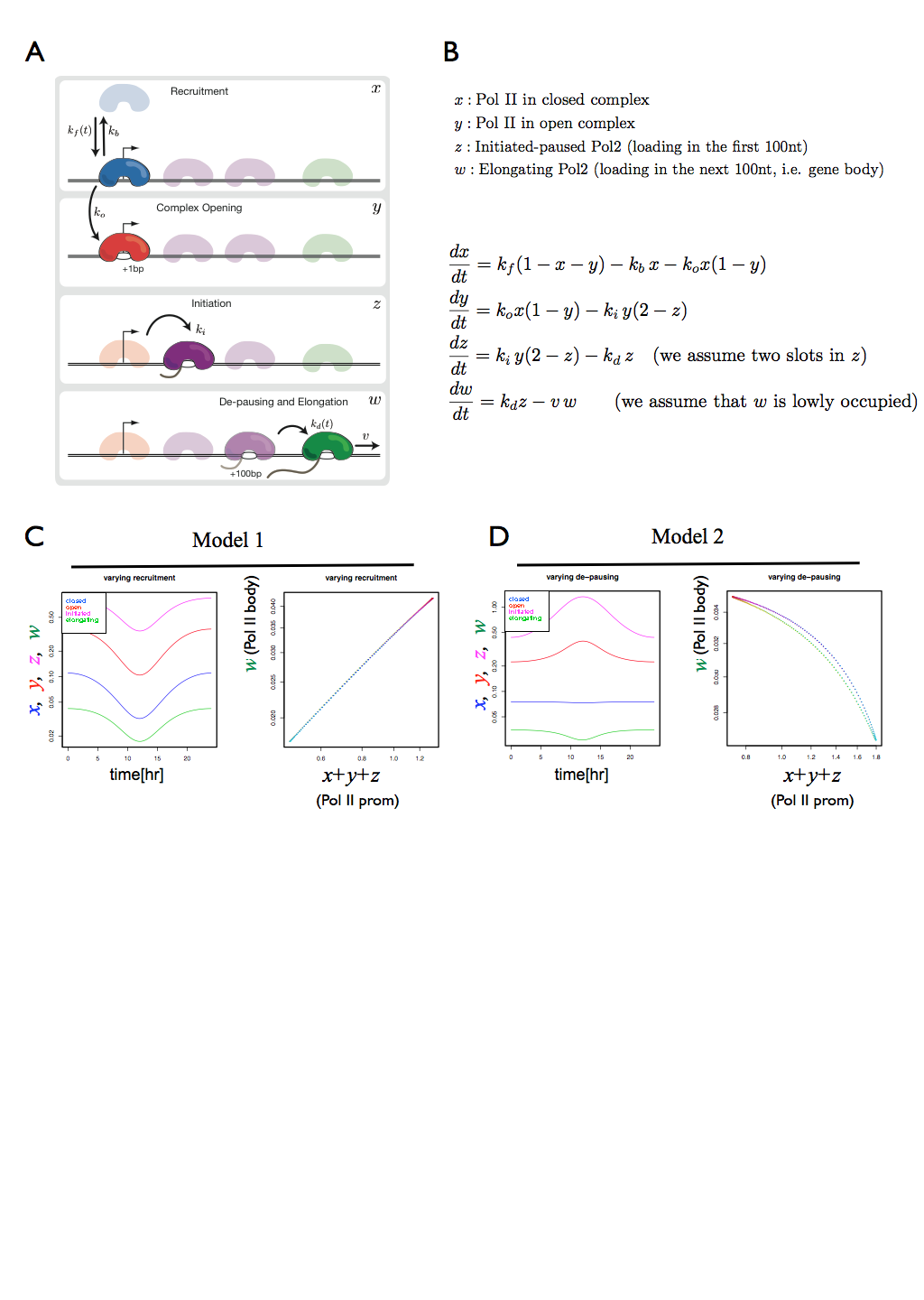

Supplement: Figure S4 — A model predicts temporal patterns for recruitment- or initiation-regulated rhythmic transcription. (A) Temporal variation of Pol II occupancy at promoter proximal positions (x,y,z) and gene bodies (w) as predicted by a simplified model of transcription. The model describes the (reversible) recruitment of polymerases to promoters, the (irreversible) transition to the open complex, the (irreversible) promoter escape, and the (irreversible) transition from a pausing to an elongating state, after which the polymerases travel to the end of the transcript. We investigate the two scenarios in which either the recruitment or the elongation rates are subject to cyclic circadian variation. (B) The mathematical model of transcription and its parameters: kf (forward recruitment rate), kb (backward recruitment rate), ko (isomerization rate), ki (promoter escape rate), kd (de-pausing rate), v (elongation rate). (C) Simulated temporal variation of occupancy in promoter proximal positions (x,y,z) and gene body (w) when the recruitment rate is varied in a circadian manner. (D) Idem when the de-pausing rate is varied in a circadian manner. The numerical values used in the simulation are kf = 1/min; kb = 0; ko = 0.1/s; ki = 1/min; kd = 1/min; v = 1/(6 s). (TIFF) [file pbio.1001442.s004.tiff]

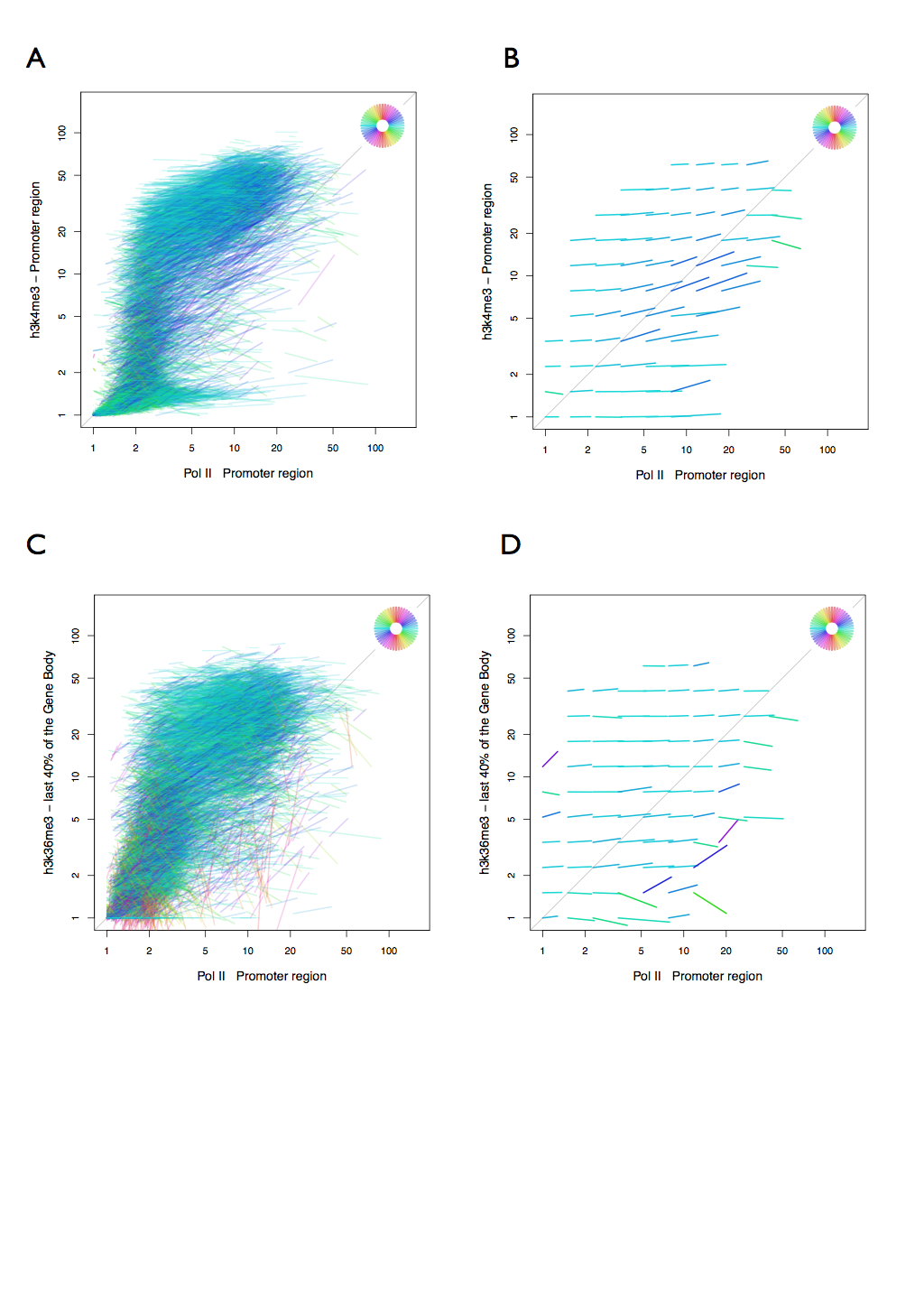

Supplement: Figure S5 — Temporal relationship of H3K4me3 and H3K36me3 marks with Pol II promoter occupancy. (A and C) Each gene is represented by a line indicating the average orientation and amplitude of changes during a diurnal cycle. Orientation is also indicated by the color (key in upper right corner). (B and D) Binned representation of (A) and (C), respectively. Panel B is identical to Figure 4F and reproduced here for comparison. (TIFF) [file pbio.1001442.s005.tiff]

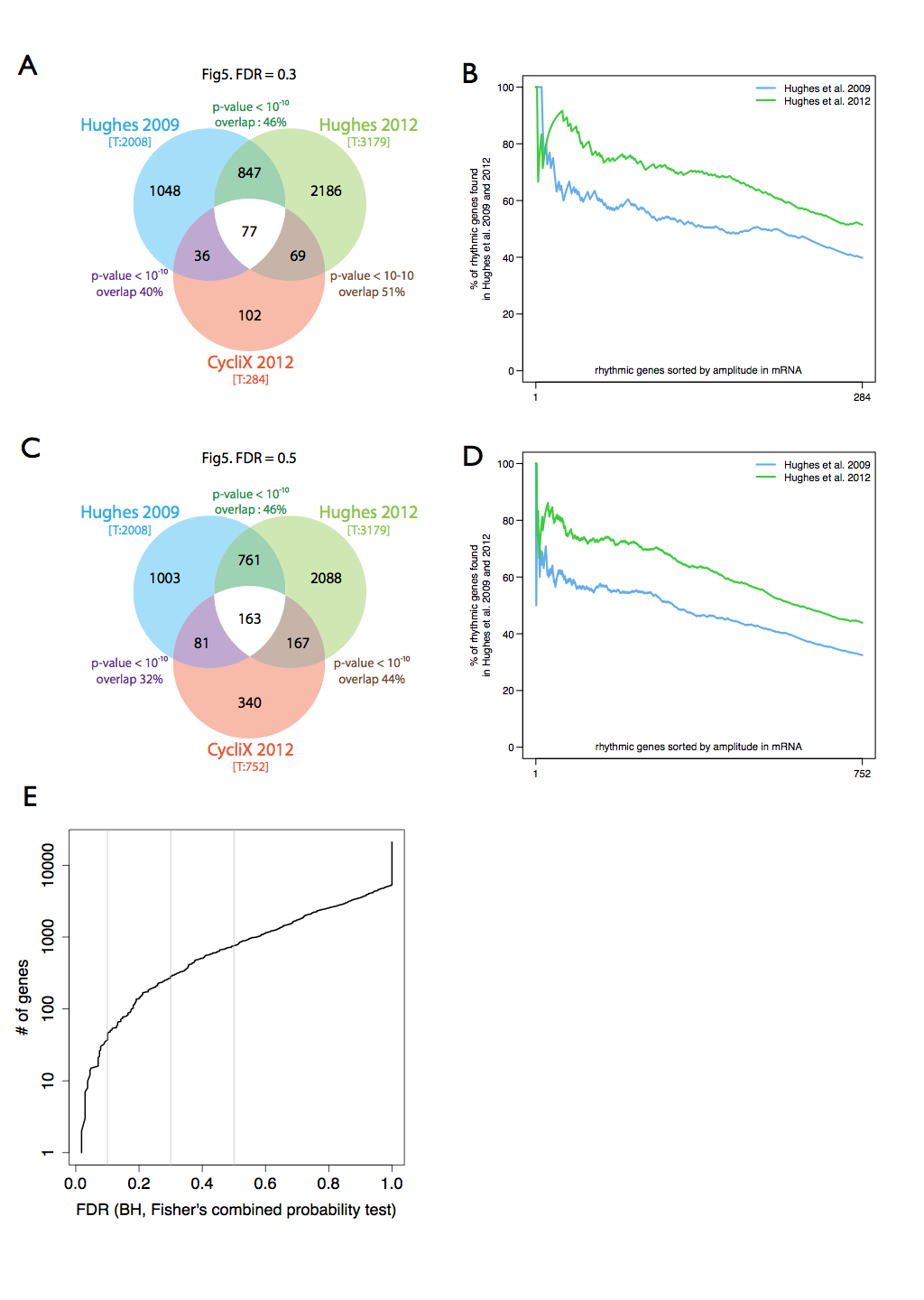

Supplement: Figure S6 — Comparison of gene selection with the Hughes et al. 2009 [41] and Hughes et al. 2012 [43] gene sets. (A) Venn diagram showing the intersection between our gene set (Figure 5, n = 284, red) and the rhythmic transcripts in Hughes et al. 2009 (blue) and Hughes et al. 2012 (green). In all pairwise comparisons, the percentage overlaps refer to the smallest of the two sets. Indicated p values for the overlaps are computed using the hypergeometric test. The gene sets and overlaps are given in Table S4. From the Hughes et al. 2009 and 2012 datasets, we only considered genes that were also measured on our arrays. Matching was done using the gene symbol. (B) The overlap is stratified according to decreasing mRNA amplitudes (peak to trough). (C–D) Idem for our less stringent gene set (Figure S7, n = 752, red). (E) Number of genes in the set as a function of the q-values (Fisher combined probability test with Benjamini-Hochberg FDR correction). (TIFF) [file pbio.1001442.s006.tiff]

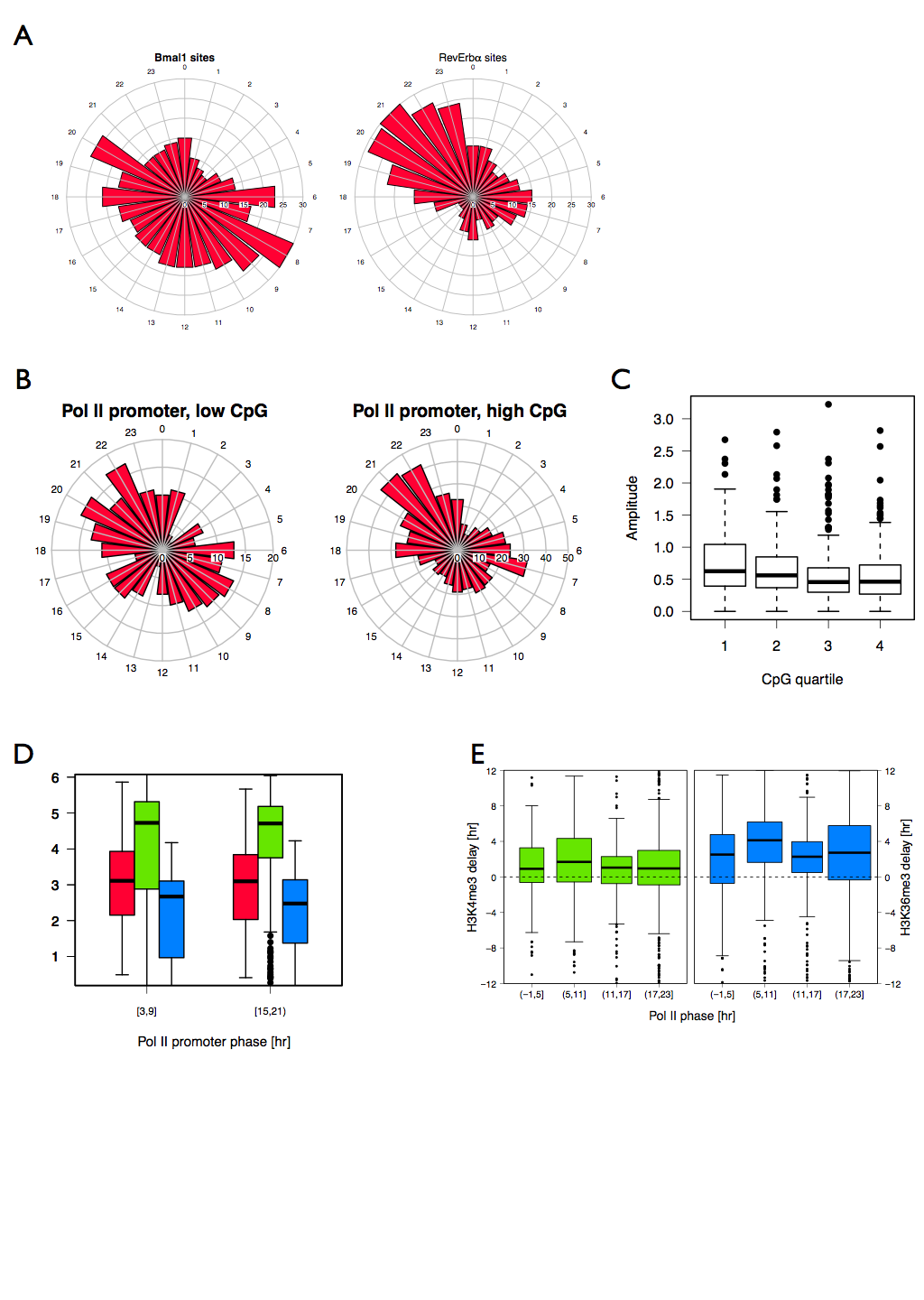

Supplement: Figure S7 — Stratification of rhythmically transcribed genes into subgroups. (A) Transcription phases for the 400 strongest BMAL1 sites in Rey et al. 2011 [11] show a maximal phase at ZT8, while the 400 strongest REVERBα sites from Cho et al. 2012 [14] show a peak at ZT21. (B) The rhythmic gene set in Figure S7A split into low and high CpG promoters show similar phase distributions. (C) The rhythmic gene set in Figure S7A split into low and high CpG promoters show that high CpG island promoters have slightly lower amplitudes. (D) Mean Pol2 (red), H3K4me3 (green), and H3k36me3 (green) for genes transcribed in the morning phase (phase interval from ZT3 to ZT9) and evening phase (phase interval from ZT15 to ZT21) show no marked difference. (E) Phase delays between H3k4me3 and H3K36me3 compared to Pol II signals are not correlated with the peak time of Pol II (bins are indicated on the x-axis). (TIFF) [file pbio.1001442.s007.tiff]

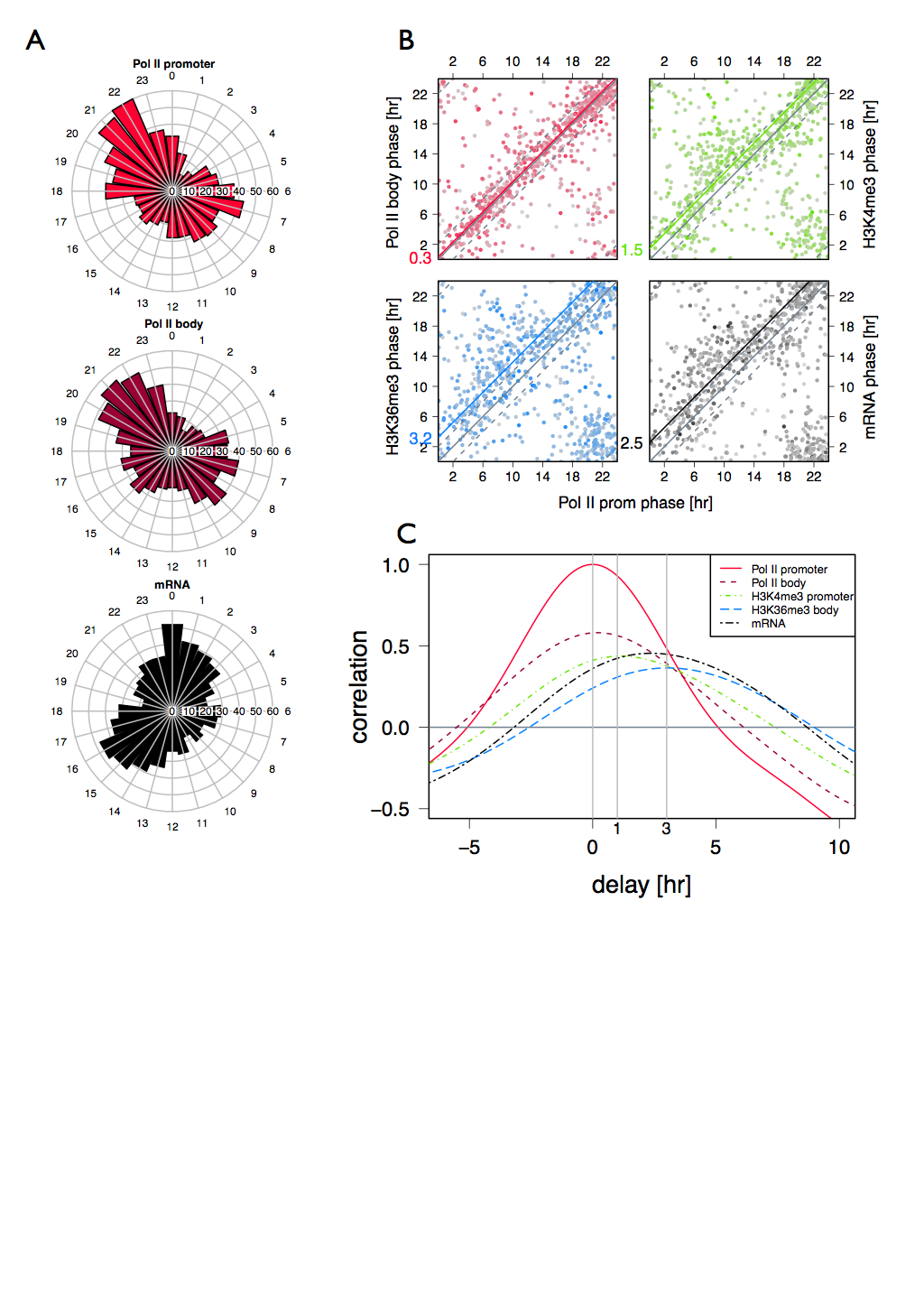

Supplement: Figure S8 — Temporal relationships of Pol II, H3K4me3, H3K36me3 profiles, and mRNA accumulation in mouse liver. Idem as Figure 5 with an extended selection of genes (n = 752, p<0.018, FDR = 0.5). (TIFF) [file pbio.1001442.s008.tiff]

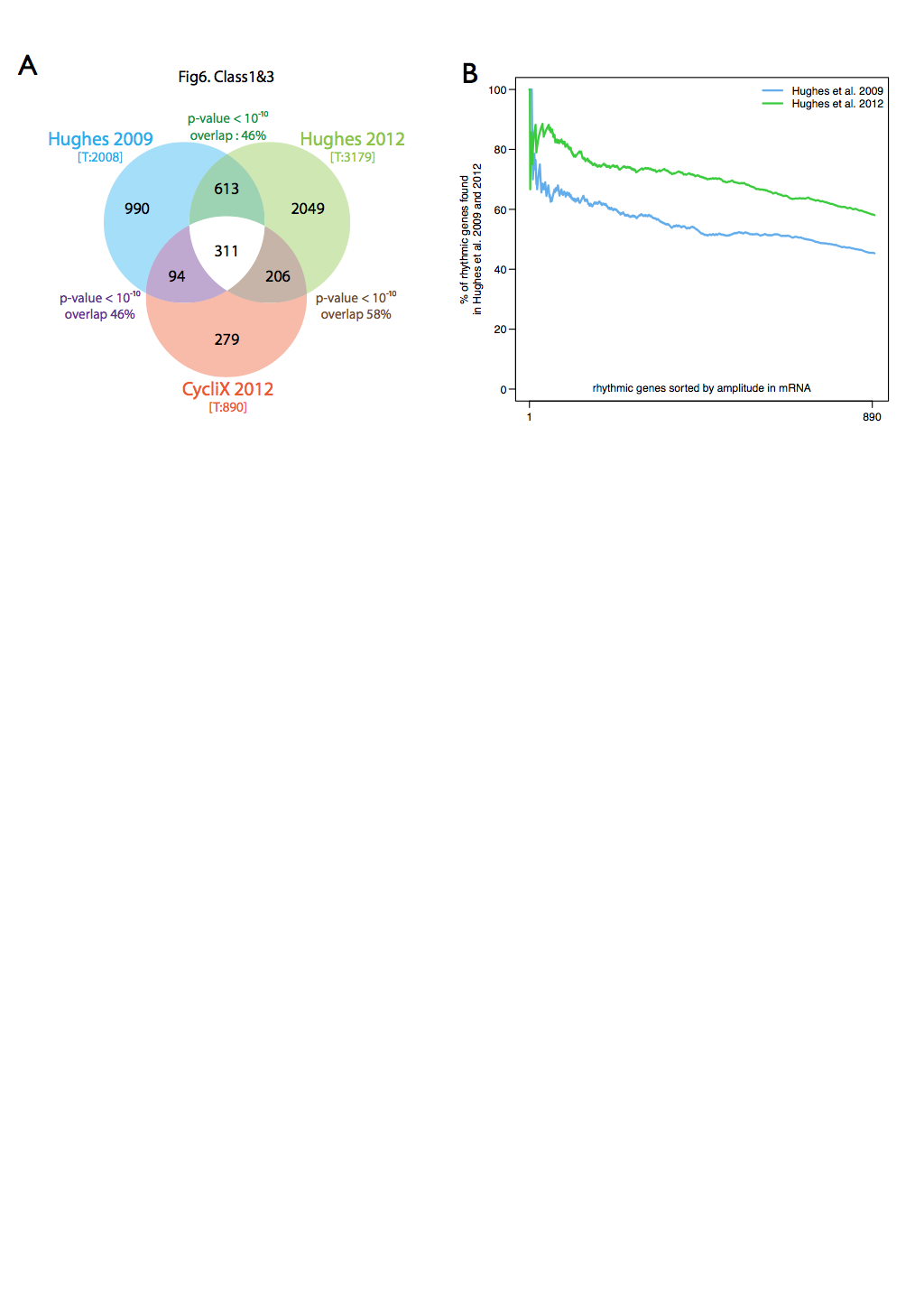

Supplement: Figure S9 — Comparison of class 1 and class 3 genes with the Hughes et al. 2009 [41] and Hughes et al. 2012 [43] gene sets. (A) Venn diagram showing the intersection between the genes of class 1 or 3—that is, transcripts that show diurnal variations of mRNA (Figure 6, n = 892, red) and the rhythmic transcripts in Hughes et al. 2009 (blue) and Hughes et al. 2012. In all pairwise comparisons, the percentage overlaps refer to the smallest of the two sets. Indicated p values for the overlaps are computed using the hypergeometric test. The gene sets and overlaps are given in Table S4. (B) The overlap is stratified according to decreasing mRNA amplitudes (peak to trough). (TIFF) [file pbio.1001442.s009.tiff]

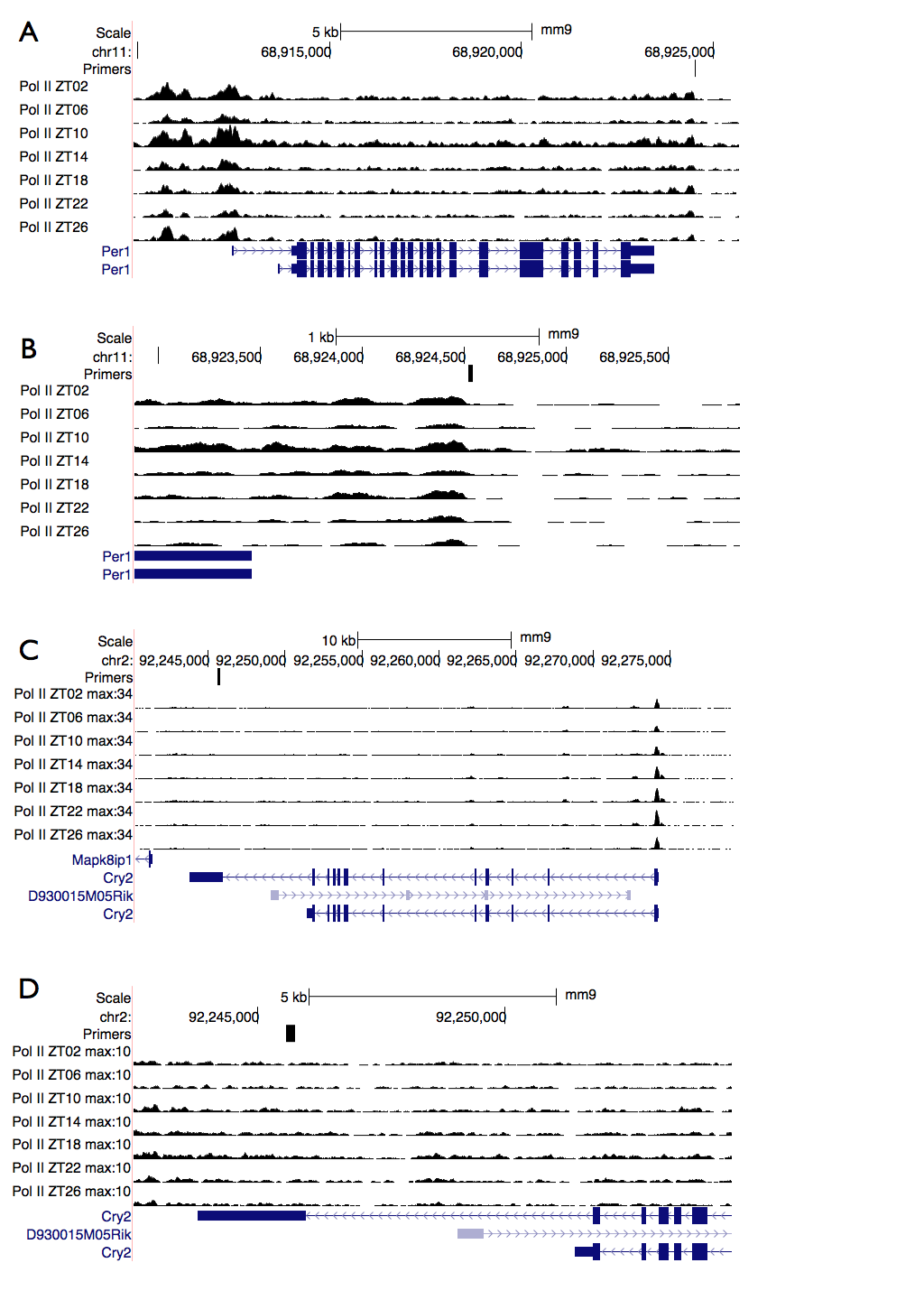

Supplement: Figure S10 — Pol II occupancy profiles at the Per1 and Cry2 genes. (A) Profile of Per1, entire locus. (B) Profile of Per1, region around the PAS. (C) Profile of Cry2, entire locus. (D) Profile of Cry2, region around the PAS. The location of ChIP-qPCR primers used in (Padmanhaban et al., Science 2012 [19]) is indicated below the chromosome coordinates. (TIFF) [file pbio.1001442.s010.tiff]

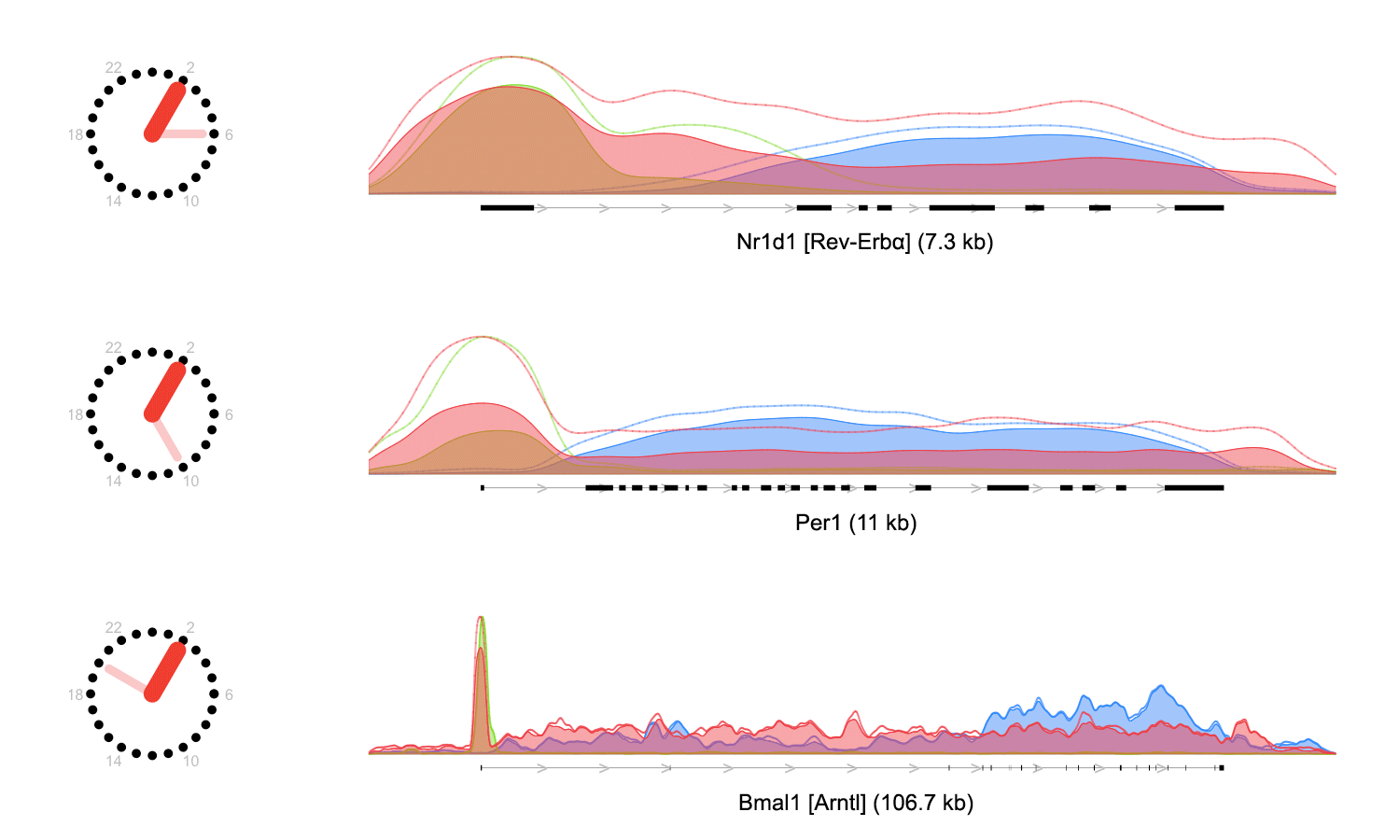

Supplement: Movie S1 — Animated profiles for the Bmal1, Reverbα (Nr1d1), and mPer1 genes for the Pol2 (red) occupancy and H3K4me3 (green) and H3K36me3 (blue) marks. The data were interpolated in time using spline interpolation (every 30 min). (GIF) [file pbio.1001442.s011.gif]

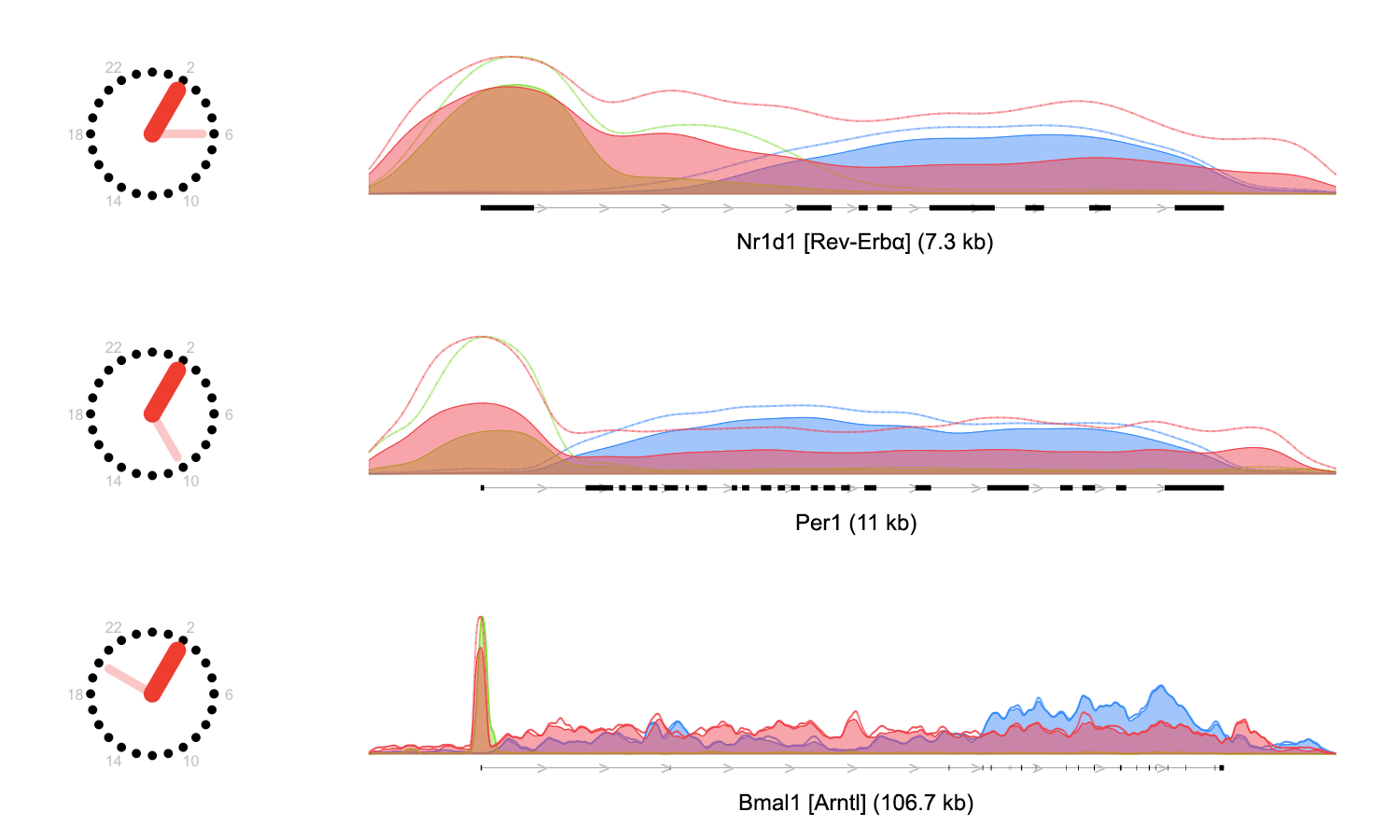

Supplement: Movie S2 — Idem as Movie S1 with only the measured time points (no interpolation). (GIF) [file pbio.1001442.s012.gif]
